# Current prevalence pattern of tobacco smoking in Nigeria: a systematic review and meta-analysis

**DOI:** 10.1186/s12889-019-8010-8

**Published:** 2019-12-21

**Authors:** Davies Adeloye, Asa Auta, Ademola Fawibe, Muktar Gadanya, Nnenna Ezeigwe, Rex G. Mpazanje, Mary T. Dewan, Chiamaka Omoyele, Wondimagegnehu Alemu, Michael O. Harhay, Isaac F. Adewole

**Affiliations:** 1 Centre for Global Health, Usher Institute, University of Edinburgh, 30 West Richmond street, Edinburgh, EH8 9DX UK; 2 RcDavies Evidence-based Medicine, Lagos, Nigeria; 3 School of Pharmacy and Biomedical Sciences, University of Central Lancashire, Fylde Road, Preston, UK; 4 Department of Medicine, University of Ilorin, Ilorin, Nigeria; 5 Department of Community Medicine, Aminu Kano Teaching Hospital, Bayero University, Kano, Nigeria; 6 Federal Ministry of Health, Abuja, Nigeria; 7 WHO Nigeria Country Office, Abuja, Nigeria; 8 International Health Consultancy, LLC, Atlanta, GA USA; 9 Department of Biostatistics, Epidemiology and Informatics, Perelman School of Medicine University of Pennsylvania, Philadelphia, PA USA; 10 Palliative and Advanced Illness Research (PAIR) Center, Perelman School of Medicine, University of Pennsylvania, Philadelphia, PA USA; 11 College of Medicine, University of Ibadan, Ibadan, Nigeria

**Keywords:** Smoking, Tobacco, Prevalence, Non-communicable diseases, Risk, Nigeria

## Abstract

**Background:**

National smoking cessation strategies in Nigeria are hindered by lack of up-to-date epidemiologic data. We aimed to estimate prevalence of tobacco smoking in Nigeria to guide relevant interventions.

**Methods:**

We conducted systematic search of publicly available evidence from 1990 through 2018. A random-effects meta-analysis and meta-regression epidemiologic model were employed to determine prevalence and number of smokers in Nigeria in 1995 and 2015.

**Results:**

Across 64 studies (*n* = 54,755), the pooled crude prevalence of current smokers in Nigeria was 10.4% (9.0–11.7) and 17.7% (15.2–20.2) for ever smokers. This was higher among men compared to women in both groups. There was considerable variation across geopolitical zones, ranging from 5.4% (North-west) to 32.1% (North-east) for current smokers, and 10.5% (South-east) to 43.6% (North-east) for ever smokers. Urban and rural dwellers had relatively similar rates of current smokers (10.7 and 9.1%), and ever smokers (18.1 and 17.0%). Estimated median age at initiation of smoking was 16.8 years (IQR: 13.5–18.0). From 1995 to 2015, we estimated an increase in number of current smokers from 8 to 11 million (or a decline from 13 to 10.6% of the population). The pooled mean cigarettes consumption per person per day was 10.1 (6.1–14.2), accounting for 110 million cigarettes per day and over 40 billion cigarettes consumed in Nigeria in 2015.

**Conclusions:**

While the prevalence of smokers may be declining in Nigeria, one out of ten Nigerians still smokes daily. There is need for comprehensive measures and strict anti-tobacco laws targeting tobacco production and marketing.

## Introduction

Smoking is a leading cause of preventable deaths and morbidity, linked to high burden of lung cancer, chronic obstructive pulmonary disease (COPD), ischemic heart diseases and stroke [1–3]. It accounts for more than 7 million deaths annually with about 10% of these resulting from second-hand smoke [2]. There are around 1.1 billion smokers worldwide and about 80% of these live in low- and middle-income countries (LMICs), where more than two-thirds of smoking-related deaths occur [2].

Though global current smoking rates among adults decreased from 23.5 to 20.7% between 2007 and 2015 [4], this reduction was largely due to the declining smoking rates in Northern and Western Europe, North America and the Western Pacific regions [3, 4], where considerable measures have been implemented to tackle tobacco smoking. Conversely, smoking rate appears to be increasing in the Middle East and Africa [4]. For example, in sub-Saharan Africa, the consumption of tobacco increased by 57% between 1990 and 2009 [5]. A recent analysis of the Demographic Health Survey data of 30 sub-Saharan African countries revealed higher smoking rates, with prevalence as high as 37.7% among men in Sierra Leone [6].

Nigeria is the most populous country in Africa and has one of the leading tobacco markets in Africa, with over 18 billion cigarettes sold annually costing Nigerians over US$ 931 million [7, 8]. Following the 2003 World Health Organization (WHO) Framework Convention on Tobacco Control (FCTC) [2], Nigeria ratified the convention agreement in 2005, and in 2015 signed into law the National Tobacco Control (NTC) Act that regulates all aspects of tobacco control including advertising, packaging, and smoke-free areas [2]. Despite these initiatives, some reports suggest the prevalence of smoking in the country is rising at about 4% per year [8].

The WHO estimated about 13 million smokers in Nigeria in 2012 [7], with over 16,000 deaths attributable to smoking [9]. Increased commerce by international tobacco companies and the relative role they play in economic growth may have contributed to a rise in smoking rates [8, 10]. Although, some national estimates of smoking prevalence have been reported [11, 12], the exact number of smokers remains debated, which possibly hinders health policy. Concerns over current estimates include varying case definitions, representativeness of study samples or data, and poor study designs. We therefore conducted a comprehensive systematic search of the literature and synthesized data based on standard case definitions to estimate national and sub-national prevalence of smoking in Nigeria.

## Methods

This is a review of publicly available studies and conducted as part of series on the epidemiology of non-communicable diseases (NCDs) in Nigeria. Methods have been described in detail in previous studies [13–16]. No ethical approval was required. Study was conducted in line with the PRISMA (Preferred Reporting Items for Systematic Reviews and Meta-Analyses) guidelines [17].

### Search strategy

We searched MEDLINE, EMBASE, Global Health and Africa Journals Online (AJOL) on 31 January 2019. We initially searched for epidemiological studies on smoking in Nigeria and sourced for unpublished reports (or studies) from Google searches and Google Scholar. We included studies that were (i) population-based, (ii) reporting on the prevalence of smoking (current or ever) in a Nigerian setting, and (iii) published on or after 01 January 1990. Search terms are presented in the Additional file 1.

### Case definitions

We selected studies that defined smoking as “smoking of tobacco products, be it cigarettes, bidi, cigar, hookah, pipe, or other related manufactured products and hand rolled stuffs”. We defined current smoker as someone who smokes every day, or some days in the last 30 days preceding an interview. An ex-smoker (or former smoker) is someone who was an every-day smoker or has smoked at least 100 cigarettes in his or her lifetime but has currently quit smoking [2, 18, 19]. An ever smoker is defined as anyone who has quit smoking (smoked at least 100 cigarettes in their lifetime) or currently smokes. This describes life-time smoking status and satisfies the definition of either a current or former smoker [19, 20].

### Data extraction

DA and AA independently reviewed and assessed studies using a pre-defined guideline to ensure consistency in studies’ selection (disagreements were resolved by consensus). From each study, we extracted number of smokers, sample size, mean (or median) age, and estimated prevalence of smoking (and confidence intervals (CI). These were matched to the study period, site, geopolitical zone and setting, respectively. Quality of studies was assessed during data extraction, adapting a previously used guideline [21–24]. This was based on representativeness of the sample, appropriate design and analysis, and standard case definitions, with each study graded as high, moderate, or low (Additional file 1).

### Data analysis

We employed a random-effects meta-analysis, using the DerSimonian and Laird Method [25], to combine individual study estimates and generate national and sub-national pooled estimates of the prevalence of tobacco smoking in Nigeria. Assuming a binomial (or Poisson) distribution, we estimated standard errors from crude prevalence and sample. Heterogeneity was identified from subgroup analyses, and assessed using I-squared (*I*^*2*^) statistics. To show trends and changes in smoking prevalence in the country, a meta-regression model accounting for the study period, and age was developed. Age-adjusted prevalence estimates were generated from the model for years 1995 and 2015. These were employed to estimate the absolute number of current and ever smokers in Nigeria based on the United Nations population (five-year age groups) for Nigeria for the two years [26]. This model has been described in detail in previous studies [13–16]. All statistical analyses were conducted on Stata (Stata Corp V.14, Texas, USA).

## Results

### Search results

**Fig. 1:**
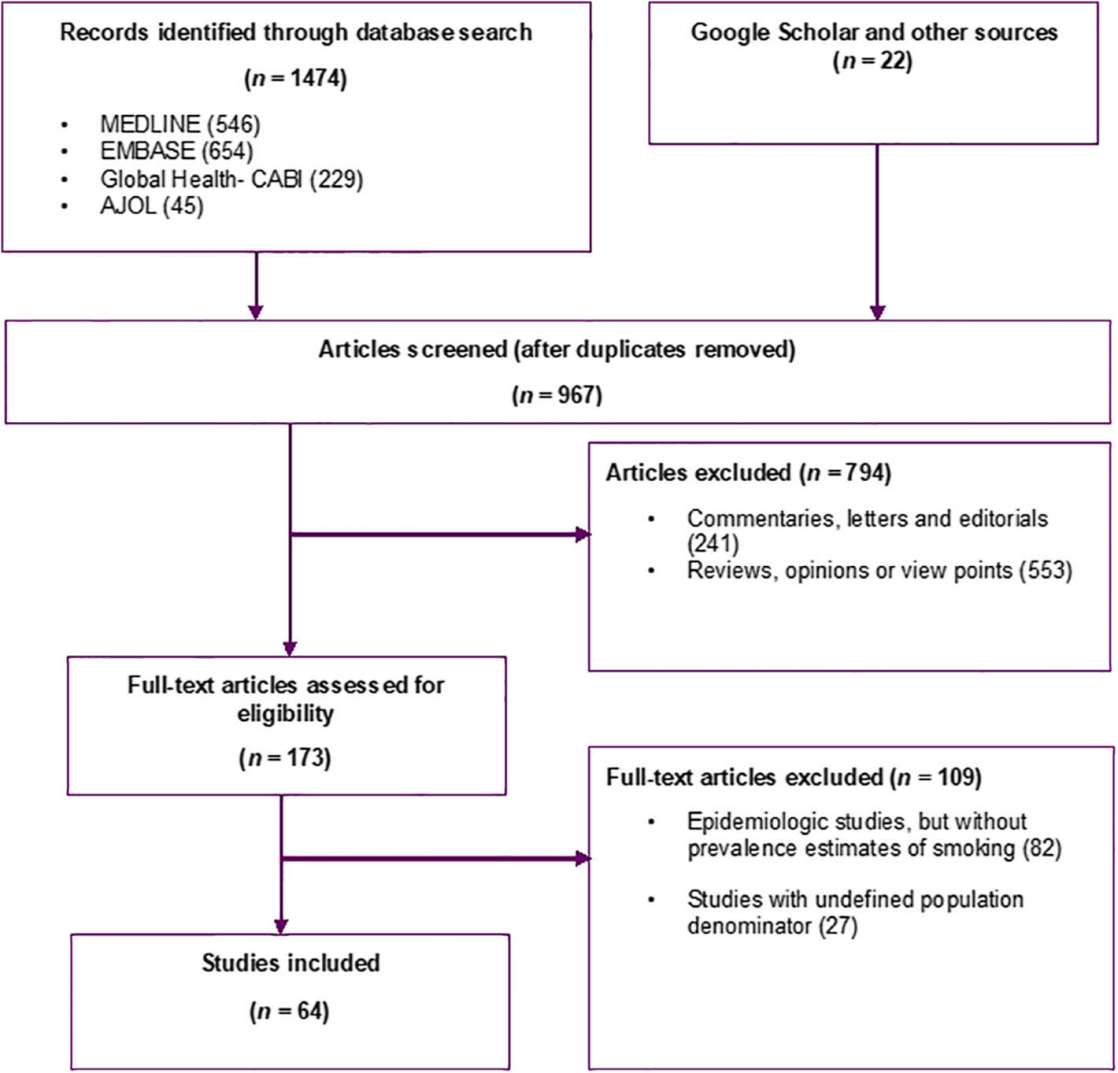
Flow chart of selection of studies on tobacco smoking in Nigeria

A total of 1474 records were retrieved from the databases – 546 studies in MEDLINE, 654 in EMBASE, 229 in Global Health, and 45 in AJOL. Twenty-two studies were identified from additional searches. We screened 967 titles were for relevance (i.e. epidemiologic studies on smoking in Nigeria) after removing duplicates, with 794 articles excluded. Abstracts and full-texts of the remaining 173 articles were accessed and screened. We retained 64 studies for synthesis (Fig. 1).

### Study characteristics

The 64 studies covered the six geopolitical zones of Nigeria (Table 1). The South-west returned 40.6% (26 studies) of all selected articles, followed by South-south (19%, 12 studies), and South-east and North-central (13%, 8 studies). The North-west was covered in four studies (6.3%), while the North-east had the lowest coverage with two studies (3.1%). Most studies (77.1%, 43 studies) were conducted in urban settings, while rural settings had 10 studies (15.6%), and 11 studies (17.2%) were from mixed urban and rural settings. Using our quality criteria, 25 studies (39%) were rated as high quality, and 39 (61%) rated as moderate. All studies were conducted under one year, with study year ranging from 1990 to 2017. The total population covered from all selected studies was 54,755, with the mean (or median) age of samples ranging from 15 to 55.5 years (Table 1 and Additional file 1). Heterogeneity was high across studies, with I-squared (*I*^*2*^) estimated at 98.2% (*P* <  0.001). This was generally high (I^2^> 95%) across subgroups (ie. sex, geopolitical zones, geographical settings) (Table 2).

**Table 1:** Characteristics of studies on prevalence of tobacco smoking in Nigeria

Author	Study period	Location	Geopolitical zone	Study design	Study setting	Sample	Current smokers (%)	Ever smokers (%)	Quality
Obaseki et al. [27]	2012	Ile-Ife, Osun State	South-west	Population-based cross-sectional study	Rural	1169	2.3	10.5	Moderate
Desalu et al. [28]	2009	Ido-Ekiti, Ekiti State	South-west	Population-based cross-sectional study	Rural	385	2.6	11.9	High
Harris-Eze [29]	1992	Ibadan, Oyo State	South-west	Cross-sectional study (soldiers)	Semi-urban	805	15.9	34.8	Moderate
Ozoh et al. [30]	2012	Idi-Araba, Lagos State	South-west	Cross-sectional study (long distance drivers)	Urban	412	1.5	13.8	Moderate
Arute et al. [31]	2015	Abraka, Delta State	South-south	Population-based cross-sectional study	Semi-urban	400	3.5	7.0	Moderate
Abiola et al. [32]	2014	Mushin, Lagos State	South-west	Population-based cross-sectional study	Urban	402	14.7	–	Moderate
Adebiyi et al. [33]	2009	Kajola, Oyo State	South-west	Descriptive cross-sectional study	Rural	215	11.6	20.5	High
Adepoju et al. [34]	2011	Osogbo, Osun State	South-west	Population-based cross-sectional study	Semi-urban	759	8.7	22.0	Moderate
Agaba et al. [35]	2014	Jos, Plateau State	North-central	Descriptive cross-sectional study	Urban	883	2.9	–	High
Agaku et al. [36]	2011	Makurdi, Benue State	North-central	Population-based cross-sectional study	Urban	536	–	27.4	High
Aina et al. [37]	2007	Idi-Aaraba, Lagos State	South-west	Cross-sectional study (healthcare students)	Urban	408	3.9	6.1	Moderate
Azodo et al. [38]	2011	Abuja, Nassarawa & Kano	North-central, North-west	Descriptive cross-sectional study (prison officials)	Urban	146	26.7	–	Moderate
Awopeju et al. [39]	2012	Ile-Ife, Osun State and Idi-Araba, Lagos State	South-west	Descriptive cross-sectional study (healthcare students)	Mixed	675	5.0	17.9	Moderate
Anyanwu et al. [40]	2015	Abakaliki, Ebonyi State	South-east	Population-based cross-sectional study	Urban	620	14.4	–	High
Akinbodewa et al. [41]	2014	Akure & Ondo, Ondo State	South-west	Descriptive cross-sectional study	Mixed	1183	–	4.5	High
Babatunde et al. [42]	2016	Ilorin, Kwara State	North-central	Population-based cross-sectional study	Urban	2000	13.6	–	Moderate
Babatunde et al. [43]	2011	Ido-Ekiti, Ekiti State	South-west	Descriptive cross-sectional study	Semi-urban	300	13.7	–	Moderate
Dania et al. [44]	2015	Yaba, Lagos State	South-west	Descriptive cross-sectional study	Urban	250	1.2	9.6	High
Desalu et al. [45]	2007	Yola, Adamawa State	North-east	Population-based cross-sectional study	Semi-urban	1793	31.9	37.9	High
Desalu et al. [46]	2009	Ilorin, Kwara State	North-central	Population-based cross-sectional study	Urban	472	8.1	12.5	High
Ebirim et al. [47]	2013	Owerri, Imo State	South-east	Descriptive cross-sectional study	Urban	944	11.2	15.3	High
Ekanem et al [48]	2008	Abuja, FCT	North-central	Population-based cross-sectional study	Urban	1399	3.5	12.0	High
Emerole et al [49]	2007	Owerri, Imo State	South-east	Descriptive cross-sectional study (University staffs)	Urban	241	3.7	–	Moderate
Fatoye & Morakinyo [50]	2001	Ilesa, Osun State	South-west	Descriptive cross-sectional study	Mixed	567	3.0	–	Moderate
Fawibe & Shittu [51]	2009	Ilorin, Kwara State	North-central	Descriptive cross-sectional study	Urban	1754	5.7	17.1	High
Hussain et al [52]	2007	Lagos State	South-west	Cross-sectional study (soldiers)	Urban	853	20.3	–	Moderate
Ibekwe [53]	2012	Oghara, Delta State	South-south	Descriptive cross-sectional study	Rural	272	15.8	–	Moderate
Makanjuola et al [54]	2004	Ilorin, Kwara State	North-central	Cross-sectional study (medical students)	Urban	961	3.2	10.5	Moderate
Morakinyo et al [55]	2003	Ibadan, Oyo State	South-west	Cross-sectional study (street children)	Urban	180	10.0	14.4	Moderate
Obot [56]	1990	Jos, Plateau State	North-central	Population-based cross-sectional study	Mixed	1271	26.8	31.5	Moderate
Odey et al [57]	2012	Calabar, Cross River State	South-south	Descriptive cross-sectional study	Urban	375	6.4	–	Moderate
Odeyemi et al [58]	2009	National	National	Descriptive cross-sectional study	Mixed	1183	17.1	26.4	Moderate
Odugbemi et al [59]	2010	Tejuosho, Lagos	South-west	Descriptive cross-sectional study	Urban	400	4.5	7.2	Moderate
Lawoyin et al [60]	1998	Idikan Ibadan, Oyo State	South-west	Population-based cross-sectional study	Rural	2144	18.5	–	Moderate
Ige et al [61]	2013	Ibadan, Oyo State	South-west	Descriptive cross-sectional study	Urban	525	1.9	–	High
Ugwuja et al [62]	2008	Abakaliki, Ebonyi State	South-east	Cross-sectional study (civil servants)	Urban	205	5.9	–	Moderate
Odukoya et al [63]	2011	Lagos State	South-west	Descriptive cross-sectional study	Urban	989	9.6	–	Moderate
Okagua et al [64]	2015	Port-Harcourt, Rivers State	South-south	Descriptive cross-sectional study	Urban	1120	3.3	7.1	High
Oladapo et al [65]	2015	Egbeda, Oyo State	South-west	Descriptive cross-sectional study	Rural	2000	1.7	–	High
Onofa et al [66]	2016	Abeokuta, Ogun State	South-west	Descriptive cross-sectional study	Urban	1233	–	14.4	Moderate
Onyeonoro et al [67]	2015	Umuahia, Abia State	South-east	Population-based cross-sectional study	Semi-urban	2983	13.0	–	Moderate
Oshodi et al [68]	2008	Surulere, Lagos State	South-west	Descriptive cross-sectional study	Urban	366	3.0	5.2	Moderate
Owonaro & Eniojukan [69]	2015	Amassoma, Bayelsa State	South-south	Descriptive cross-sectional study	Urban	254	55.5	64.6	Moderate
Owonaro & Eniojukan [70]	2015	Opokuma, Bayelsa State	South-south	Descriptive cross-sectional study	Rural	252	10.7	20.2	Moderate
Ozoh et al [71]	2014	Lagos mainland, Lagos State	South-west	Cross-sectional study (commercial drivers)	Urban	500	32.0	57.2	Moderate
Ozoh et al [72]	2017	Lagos mainland, Lagos State	South-west	Cross-sectional study (long distance drivers)	Urban	414	29.7	40.8	Moderate
Raji et al [73]	2012	Sokoto, Sokoto State	North-west	Descriptive cross-sectional study	Urban	228	8.3	–	High
Raji et al [74]	2017	Sokoto, Sokoto State	North-west	Descriptive cross-sectional study	Urban	213	3.3	11.3	Moderate
Salawu et al. [75]	2009	Yola, Adamawa	North-east	Population-based cross-sectional study	Semi-urban	171	33.9	50.3	Moderate
Shehu & Idris [76]	2004	Saria, Kaduna State	North-west	Descriptive cross-sectional study	Semi-urban	350	9.4	–	High
Yisa et al. [77]	2009	Ibadan, Oyo State	South-west	Descriptive cross-sectional study	Urban	510	2.1	–	High
Abasiubong et al. [78]	2005	Eket, Akwa-Ibom State	South-south	Descriptive cross-sectional study	Mixed	254	34.8	–	Moderate
Gureje et al. [79, 80]	2007	National	National	Population-based cross-sectional study	Mixed	6752	4.2	17.0	High
Lasebikan et al. [81]	2016	Oyo State	South-west	Population-based cross-sectional study	Rural	1203	20.6	33.7	Moderate
Odenigbo et al. [82]	2008	Asaba, Delta State	South-south	Cross-sectional study (healthy professionals)	Semi-urban	100	2.0	–	Moderate
Forrest et al. [83]	1992	Benin, Edo State	South-south	Population-based cross-sectional study	Urban	464	–	11.5	High
Oguoma et al. [84]	2015	Kwale, Delta State	South-south	Population-based cross-sectional study	Mixed	422	3.4	11.2	High
Ezejimofor et al. [85]	2014	Niger Delta, Delta State	South-south	Community-based cross-sectional study	Rural	2028	–	16.7	High
Ezekwesili et al. [86]	2016	Anambra State	South-east	Population-based cross-sectional study	Mixed	912	3.1	–	Moderate
Ogah et al. [87]	2012	Umuahia, Abia State	South-east	Population-based cross-sectional study	Mixed	2983	–	13.3	High
Suleiman et al. [88]	2011	Amassoma, Bayelsa State	South-south	Descriptive cross-sectional study	Semi-urban	400	–	14.3	Moderate
Ugwuja et al. [89]	2015	Igbeagu, Ebonyi State	South-east	Population-based cross-sectional study	Rural	267	–	3.00	High
Wahab et al. [90]	2006	Katsina, Katsina State	North-west	Population-based cross-sectional study	Urban	300	4.7	–	High

**Table 2:** Pooled crude estimates of prevalence of smokers in Nigeria

			Both sexes		Men		Women	
			Prevalence % (95% CI)	I^2^%, P-value	Prevalence % (95% CI)	I^2^%, *P*-value	Prevalence % (95% CI)	I^2^%, P-value
Nation-wide		Current	10.4 (9.0–11.7)	98.2, < 0.001	13.4 (10.0–16.8)	98.4, < 0.001	3.6 (2.8–4.4)	95.2, < 0.001
		Ever	17.7 (15.2–20.2)	98.6, < 0.001	22.8 (17.5–28.2)	98.7, < 0.001	6.3 (4.8–7.7)	96.8, < 0.001
Geopolitical zone	North-central	Current	10.3 (6.0–14.3)	98.7, < 0.001	8.1 (4.3–11.9)	94.8, < 0.001	3.6 (0.7–6.4)	97.1, < 0.001
		Ever	18.4 (12.2–24.7)	97.9, < 0.001	24.0 (13.6–34.5)	98.0, < 0.001	7.3 (5.7–8.9)	21.4, 0.280
	North-east	Current	32.1 (30.0–34.1)	0.0, 0.593	44.8 (41.7–47.8)	0.0, 0.323	18.6 (16.2–21.1)	0.0, 0.444
		Ever	43.6 (31.5–55.7)	89.6, < 0.001	54.7 (51.6–57.7)	0.0, 0.619	28.9 (11.9–45.9)	86.9, < 0.001
	North-west	Current	5.4 (3.7–7.2)	55.9, 0.078	9.5 (7.0–11.9)	0.0, 0.507	4.3 (1.0–7.6)	77.7, 0.011
		Ever	12.4 (7.9–16.9)	84.1, < 0.001	29.2 (25.3–33.1)	–	–	–
	South-east	Current	8.6 (4.1–13.0)	97.3, < 0.001	15.8 (11.6–19.9)	0	13.0 (9.3–16.7)	0
		Ever	10.5 (3.7–17.4)	97.7, < 0.001	26.8 (24.5–29.1)	–	–	–
	South-south	Current	13.0 (8.7–17.3)	97.7, < 0.001	10.2 (5.9–14.5)	74.4, 0.008	1.8 (0.6–3.0)	49.3, 0.116
		Ever	16.9 (11.4–22.3)	97.9, < 0.001	15.5 (10.7–20.2)	83.9, < 0.001	3.3 (0.7–6)	90.6, < 0.001
	South-west	Current	8.9 (6.9–11.0)	97.7, < 0.001	9.5 (6.2–12.8)	96.6, < 0.001	2.78 (1.7–3.8)	92.6, < 0.001
		Ever	17.1 (12.8–21.4)	98.8, < 0.001	15.6 (9.7–21.5)	96.6, < 0.001	7.3 (3.4–11.2)	97.4, < 0.001
Settings	Urban	Current	10.7 (8.8–12.6)	98.0, < 0.001	13.2 (9.8–16.6)	97.7, < 0.001	4.2 (3.0–5.5)	94.7, < 0.001
		Ever	18.1 (14.6–21.6)	98.6, < 0.001	20.5 (13.7–27.3)	97.8, < 0.001	6.7 (4.3–9.1)	96.0, < 0.001
	Rural	Current	9.1 (5.1–13.0)	98.7, < 0.001	15.3 (2.5–33.2)	98.9, < 0.001	7.0 (2.1–16.2)	97.7, < 0.001
		Ever	16.8 (10.8–22.8)	98.1, < 0.001	18.5 (15.9–21.1)	0	18.5 (16.4–20.7)	0
	Mixed	Current	10.2 (7.3–13.1)	98.1, < 0.001	11.8 (4.2–27.8)	99.4, < 0.001	1.0 (0.04–1.9)	61.3, 0.075
		Ever	17.3 (12.6–21.9)	98.8, < 0.001	30.3 (27.5–33.0)	85.8, < 0.001	2.6 (1.5–3.7)	90.3, < 0.001
Mean cigarette per person per day ^a^	Nation-wide		10.1 (6.1–14.2)	97.9, < 0.001	–	–	–	–

^a^absolute numbers of cigarettes consumed per person per day

### Prevalence of tobacco smoking in Nigeria

#### Current smokers

**Fig. 2:**
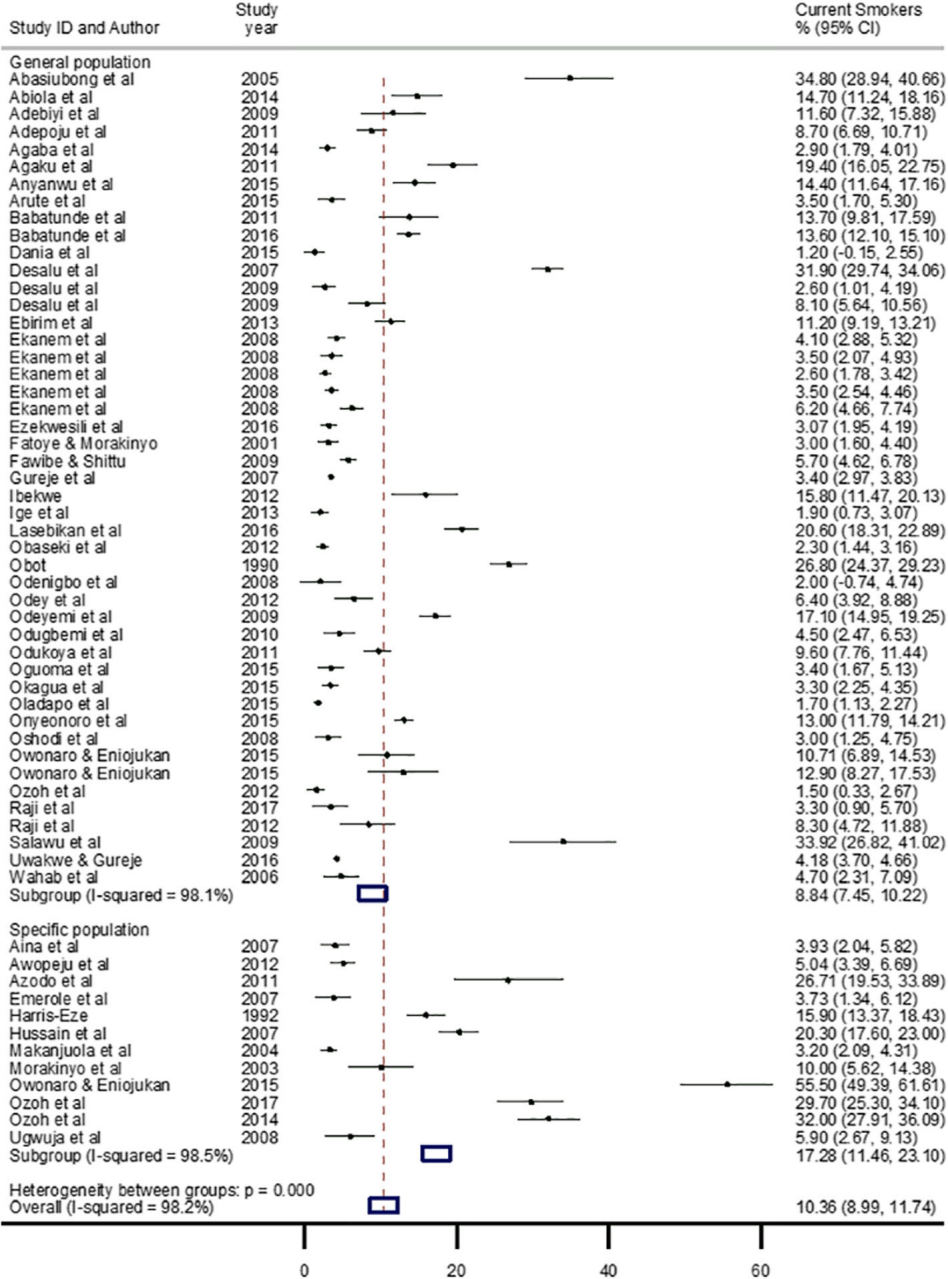
Crude prevalence rate of current smokers in Nigeria

The prevalence of current smokers ranged from 1.2% recorded in Yaba Lagos, South-west Nigeria in 2015 [44], to 55.5% in Amassoma Delta State, South-south Nigeria, also in 2015 [69]. The pooled crude prevalence of current smokers in Nigeria was 10.4% (95% CI: 9.0–11.7), with this significantly lower among women (3.6%, 2.8–4.4), compared to men (13.4%, 10.0–16.8) (Table 2). Following a sensitivity analysis, the prevalence of current smokers in the general population at 8.8% (7.5–10.2) was comparable to the overall pooled estimate (10.4%), while a higher estimate was reported among specific populations (eg. commercial drivers, soldiers, and healthcare students) at 17.3% (11.5–23.1) (Fig. 2). Across the geopolitical zones, the prevalence rate of current smokers was significantly higher in the North-east (32.1%, 30.0–34.1), compared to the other five geopolitical zones. The South-south region had a prevalence of 13.0% (8.7–17.3), North-central 10.3% (6.0–14.4), South-west 8.9% (6.9–11.0), South-east 8.6% (4.1–13.0) and North-west 5.4% (3.7–7.2) (Table 2). There was relatively no difference in the prevalence of current smokers across geographical settings, with the urban and rural settings having a prevalence of 10.7% (8.8–12.6) and 9.1% (5.1–13.0), respectively (Table 2 and Additional file 1).

#### Ever smokers

**Fig. 3:**
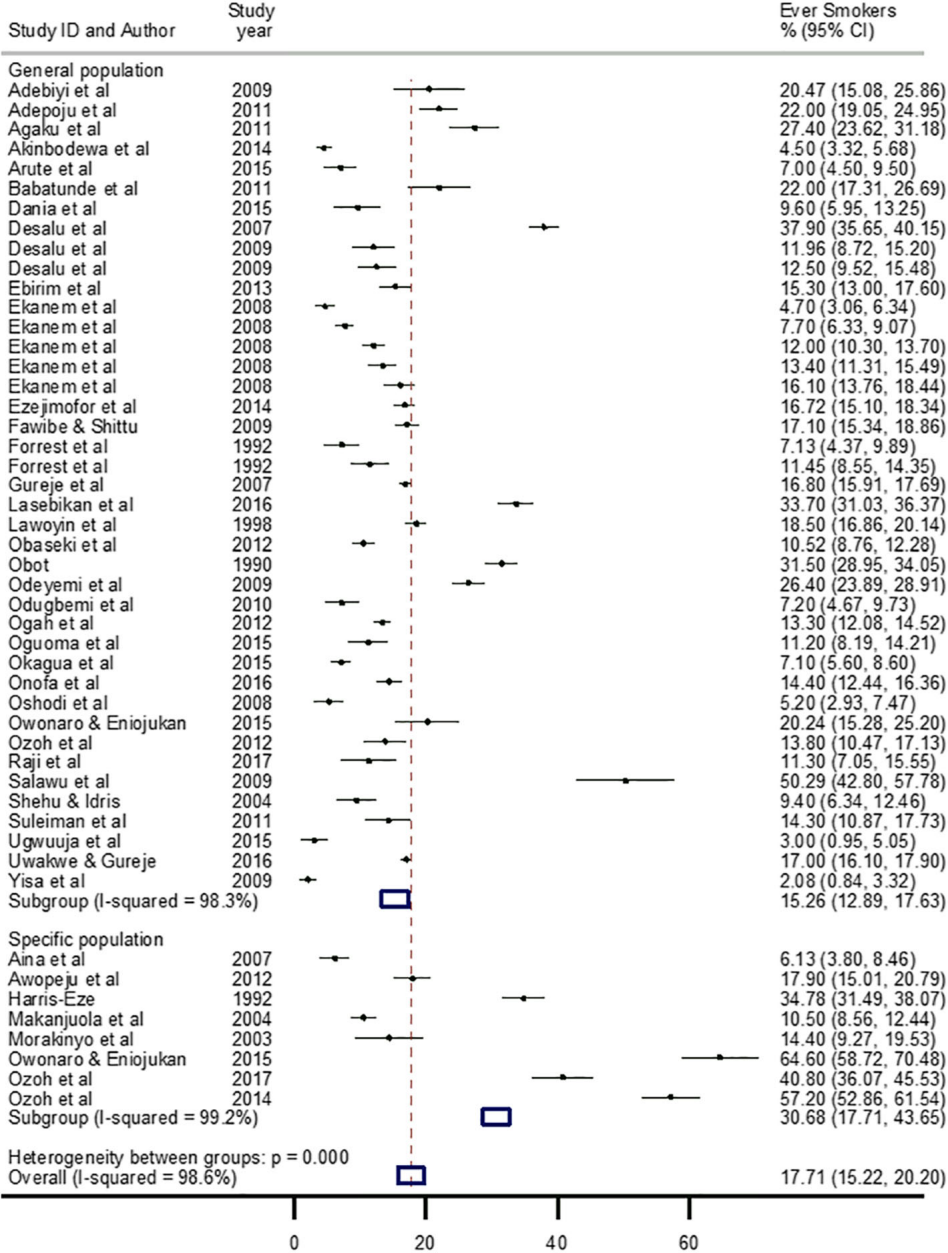
Crude prevalence rate of ever smokers in Nigeria

The lowest prevalence of ever smokers was recorded in Ibadan Oyo State, South-west Nigeria in 2009 at 2.1% [77], while the highest was reported in Amassoma Delta State, South-south Nigeria in 2015 at 64.6% [69]. The pooled crude prevalence of ever smokers (i.e. life-time prevalence of smoking) was 17.7% (95% CI: 15.2–20.2) (Table 2). As observed among current smokers, the prevalence was significantly higher among men at 22.8% (17.5–28.2), compared to women at 6.3% (4.8–7.7) (Table 2). When population characteristics were considered in the sensitivity analysis, the prevalence of ever smokers in the general population was 15.3% (12.9–17.6), which was comparable to the overall estimate (17.7%), in contrast to a relatively higher estimate among specific population groups at 30.7% (17.7–43.7) (Fig. 3). The pooled prevalence of ever smokers was highest in the North-east at 43.6% (31.5–55.7), with lowest recorded in the South-east at 10.5% (3.7–17.4) and the North-west at 12.4% (7.9–16.9). The South-south and South-west have a relatively similar pooled prevalence rates of ever smokers at 16.9% (11.4–22.3) and 17.1% (12.8–21.4), respectively. The pooled prevalence was minimally higher in urban settings at 18.1% (14.6–21.6) compared to rural settings at 16.8% (10.8–22.8) (Table 2).

#### Age at initiation of smoking

Most studies reported the mean or median age at initiation of smoking during adolescence, with this ranging from 12 years in Ibadan Oyo State, South-west Nigeria [55], to 21.9 years in Lagos Mainland, South-west Nigeria [72]. From all studies, the estimated median age at initiation of smoking was 16.75 years (interquartile range: 13.5–18.0).

### Estimated number of current and ever smokers in Nigeria

Based on the model, the age-adjusted prevalence of current smokers decreased with advancing age, while the prevalence increased with advancing age for ever smokers (Table 3). Using the United Nations demographic projections for Nigeria, we estimated about 8 million current smokers in Nigeria in 1995 among person aged 15 years or more, with this increasing to about 11 million current smokers by 2015. The age-adjusted prevalence of current smokers actually decreased from 13.0 to 10.6% over this period (Table 3). On the contrary, both the prevalence and number of ever smokers increased over the same period, from about 10.9 million (17.6%) in 1995 to 19.8 million (19.2%) in 2015 (Table 3).

**Table 3:** Absolute number of current and ever smokers in Nigeria, aged 15 years or more, 1995 and 2015

Age (years)	Current smokers				Ever smokers			
	1995		2015		1995		2015	
	*%*	*n* (000)	*%*	*n* (000)	*%*	*n* (000)	*%*	*n* (000)
15–24	13.8	1633.5	11.4	2127.4	16.1	1913.7	17.7	3291.8
20–24	13.5	1316.7	11.2	1791.6	16.5	1610.6	18.1	2897.3
25–29	13.3	1039.7	11.0	1543.5	17.0	1327.3	18.6	2608.4
30–34	13.1	861.6	10.8	1302.2	17.4	1147.4	19.0	2299.3
35–39	12.9	711.4	10.5	1051.7	17.9	988.1	19.4	1940.0
40–44	12.6	582.5	10.3	800.8	18.3	843.4	19.9	1543.4
45–49	12.4	483.1	10.1	605.9	18.7	729.1	20.3	1220.0
50–54	12.2	405.7	9.9	492.4	19.2	638.2	20.7	1035.7
55–59	12.0	321.7	9.6	399.5	19.6	527.3	21.2	877.9
60–64	11.7	245.3	9.4	312.9	20.0	418.8	21.6	718.7
65–69	11.5	177.7	9.2	234.6	20.5	316.1	22.0	563.0
70–74	11.3	116.4	9.0	163.2	20.9	215.6	22.5	409.5
75–79	11.1	64.3	8.7	94.1	21.3	124.1	22.9	246.9
80+	10.7	37.8	8.4	60.5	22.0	77.8	23.6	170.4
All	13.0	7997.4	10.6	10,980.3	17.6	10,877.3	19.2	19,822.3

Note: Estimates based on the epidemiologic modelling from all datapoints

#### Cigarettes consumed per day

**Fig. 4:**
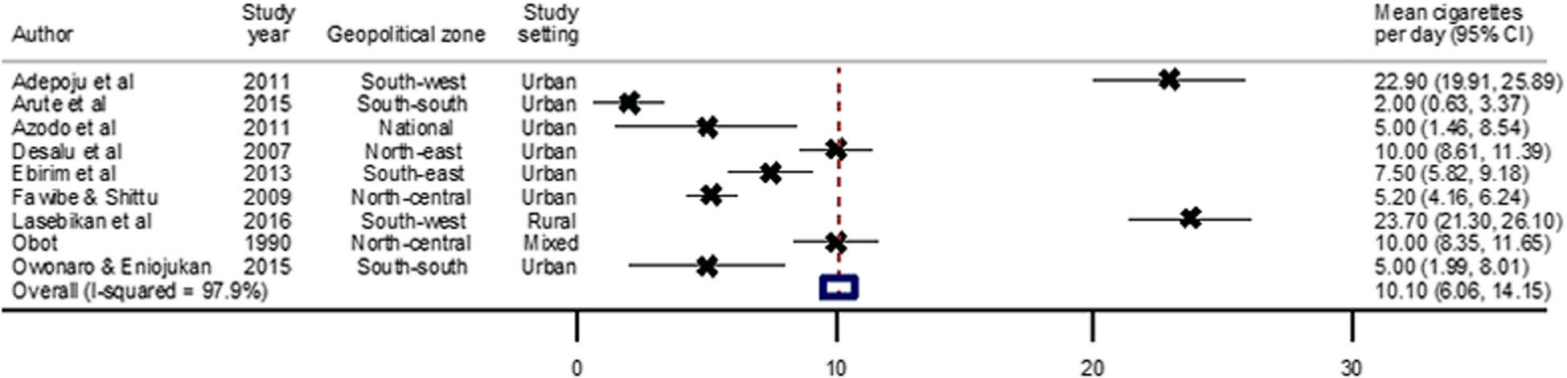
Pooled mean cigarettes consumed per person per day in Nigeria

Among current smokers, the mean cigarettes consumed per person per day ranged from 2 (1.0–3.4) recorded in a semi-urban setting in Abraka Delta State, South-south Nigeria [31], to 23.7 (21.3–26.1) in a rural area in Oyo State, Nigeria [81]. The pooled mean cigarettes consumption per person per day from all studies was 10.1 (6.1–14.2) (Table 3, Fig. 4). When considered in terms of the absolute number of current smokers in Nigeria in 2015 (11 million), this accounts for about 110 million cigarettes per day and over 40 billion cigarettes in Nigeria in 2015.

## Discussion

This study integrated smoking information from 64 moderate to high-quality studies to estimate the current prevalence of smoking in Nigeria. Although the prevalence of ever smokers increased between 1995 and 2015, we observed a decreasing prevalence of current smokers over the same period. This trend is in contrast to estimates reported, albeit based on limited data, in some countries insub-Saharan Africa, who have experienced rising smoking rates due to changing socio-economic status, rural-urban migration and increased cigarette affordability [91]. The decreasing smoking rates in Nigeria possibly reflect increased health risk awareness and better overall measures to help smokers quit in the country. For example, in a national survey, Kale and colleagues [92] reported that in the 12 months preceding their study, almost half of current smokers attempted to quit smoking, with over two-thirds of these receiving advice from care providers and counselors.

Despite the declining rates, we estimated about 11 million current smokers (10.6%) and 20 million ever smokers (19.2%) in 2015, which are still unacceptably high from an absolute perspective. In a nation-wide survey in 2012 [11], the prevalence of current smokers was 4% among adults Nigerians. This is much lower than estimated in this study, presumably due to challenges with sampling and case ascertainment. In a recent scoping exercise, Adeoye et al. [93] estimated a prevalence of current smokers at 19.7%. However, this estimate was not age-standardized, and a lower prevalence of ever smokers reported raises concerns on the quality of data. However, in 2015, the WHO reported a current smoking prevalence of about 9% among persons aged 15 years or more (17% among men and 1% among women) in Nigeria [7]. The overall prevalence and sex distribution are almost as reported in the current study. The higher smoking prevalence among men in Nigeria is well-documented [10, 93]. This perhaps represents a sustained pattern of smoking epidemic, and presents a valuable opportunity for developing effective policies and interventions learning from actions in developing countries [94, 95].

The median age at initiation of smoking in this study (16.8 years) is relatively low, reflective of a growing burden among adolescents and youths. Kale and colleagues [92] in their nation-wide survey noted that about two-thirds of the population started smoking before attaining 20 years. Adeoye et al. [93] reported lower age at initiation of 14.7 years in the country. Many have advocated for stiffer anti-tobacco laws in the country, particularly to address a growing use of tobacco products among youths [11].

The prevalence of smokers was notably higher in North-east Nigeria which may be expected given an ongoing armed conflict lasting more than a decade. Although the evidence of the association between smoking and conflict is limited and inconclusive [96], varying social situations among vulnerable populations are known to precipitate substance use [97]. With several persons displaced, children and adolescents out of school, and youths without jobs, substance use, including tobacco products, is likely to increase in these settings. Although Kale and colleagues [92] reported South-easterners as the highest consumers of tobacco products in the country, the deviance from our estimates suggests a need for more research to understand regional variations.

Although the NTC Act was signed into law in 2015 and the country has committed to the WHO FCTC since 2005 [18], Nigeria is not yet on track to achieve tobacco control targets [98]. For example, our estimates show that rural dwellers smoke almost at the same rate as urban dwellers, indicating that smoking, believed to be associated with urbanization, has gradually penetrated remote areas. Further, we estimated that current smokers consume an average of 10 cigarettes per person per day accounting for about 110 million cigarettes per day and over 40 billion cigarettes in 2015 alone. Vellios et al. [99] noted that the demand for cigarettes increased by 44% across many African countries between 1990 and 2012, with this leading to over 100% increase in cigarettes production over the same period in these countries. A thriving tobacco market raises serious public health concerns, particularly for a country with a relatively weak health system. Tobacco companies see these countries as emerging markets due to weak tobacco control regulations and several vulnerable populations [91, 94]. Careful incorporation of the WHO MPOWER package (targeted at reversing tobacco epidemic) [18] beyond the national level to state and local levels may complement successful measures like smoke-free legislation, taxes, health education and media campaigns [2, 7]. Besides, Nigeria needs to develop comprehensive surveillance systems to monitor the production, sales, and consumption of cigarettes to effectively achieve control targets [99].

The strength of this review lies in the number of studies retained (64) and population covered (54755), which spread across all geopolitical zones in the country. Herein, we have perhaps addressed an issue bordering on representativeness, which appears to be a leading concern in the understanding of the epidemiology of smoking in Nigeria [10]. We acknowledge that pooling prevalence rates from a range of studies conducted over a 27-year period (1990–2017) could affect reliability of our overall estimates; however, this was mainly done to understand the trend in smoking rates over this period, which our model and age-adjusted estimates clearly reflect (Table 3). Nonetheless, our estimates should be considered with the high heterogeneity reported. This perhaps could be due to diverse population characteristics, particularly those contributed by specific population groups. Our sensitivity analysis may have addressed this (ie. comparing general to specific populations), as excluding some of specific populations with higher prevalence of smoking could imply missing some necessary information on the use of tobacco and related products in the country. Varying study designs are also important sources of heterogeneity. Due to data limitations, we could not investigate other sources of heterogeneity, including socio-economic status, wealth index, employment status and religion. Finally, there were only two studies from the North-east, this should guide interpretation of the high estimates in the region.

## Conclusion

While the prevalence of current smokers may be declining in Nigeria, the absolute number of active smokers remain one of the highest in Africa. Economic growth, improved socio-economic status, rapid migration, and increased cigarette affordability are key factors. As rural dwellers are almost as affected as urban dwellers, careful consideration is required during programming. Comprehensive measures and strict anti-tobacco laws targeting tobacco production and marketing need to be enforced across country levels.

## Supplementary information

**Additional file 1: Table S1.** Search terms on tobacco smoking in Nigeria. **Table S2.** Quality assessment of selected studies. **Table S3.** Quality appraisal guide. **Table S4.** All extracted data employed in analysis. **Figure S1.** Crude prevalence rate of current smokers in Nigeria, by geopolitical zones. **Figure S2.** Crude prevalence rate of ever smokers in Nigeria, by geopolitical zones. **Figure S3.** Pooled mean cigarettes consumed per person per day in Nigeria. **Figure S4.** Meta-regression modelling.

## Data Availability

All data generated or analysed during this study are included in this published article [and its supplementary information files].

## Abbreviations

AJOL: Africa Journals Online
COPD: Chronic Obstructive Pulmonary Disease
FCTC: Framework Convention on Tobacco Control
LMICs: Low- and Middle-Income Countries
NCDs: Non-Communicable Diseases
NTC: National Tobacco Control
PRISMA: Preferred Reporting Items for Systematic Reviews and Meta-Analyses
WHO: World Health Organization
