# Supplementary material for: Current prevalence pattern of tobacco smoking in Nigeria: a systematic review and meta-analysis
Source: BMC Public Health. 2019 Dec 21;19:1719. doi: 10.1186/s12889-019-8010-8 (PMC6925864; doi:10.1186/s12889-019-8010-8)
Supplement: Supplementary file 1 — Additional file 1: Table S1. Search terms on tobacco smoking in Nigeria. Table S2. Quality assessment of selected studies. Table S3. Quality appraisal guide. Table S4. All extracted data employed in analysis. Figure S1. Crude prevalence rate of current smokers in Nigeria, by geopolitical zones. Figure S2. Crude prevalence rate of ever smokers in Nigeria, by geopolitical zones. Figure S3. Pooled mean cigarettes consumed per person per day in Nigeria. Figure S4. Meta-regression modelling. [file 12889_2019_8010_MOESM1_ESM.docx]

**Current prevalence pattern of tobacco smoking in Nigeria: a systematic review and meta-analysis**

**Davies Adeloye^1,2*^, Asa Auta^3*^, Ademola Fawibe^4^, Muktar Gadanya^5^, Nnenna Ezeigwe**^6^**, Rex G Mpazanje^7^, Mary T Dewan^7^, Chiamaka Omoyele^6^, Wondimagegnehu Alemu^8^, Michael O Harhay^9,10^, Isaac F Adewole^11^**

1. Centre for Global Health, Usher Institute, University of Edinburgh, UK.
2. RcDavies Evidence-based Medicine, Lagos, Nigeria.
3. School of Pharmacy and Biomedical Sciences, University of Central Lancashire, Fylde Road, Preston, UK
4. Department of Medicine, University of Ilorin, Ilorin, Nigeria
5. Department of Community Medicine, Aminu Kano Teaching Hospital, Bayero University, Kano, Nigeria
6. Federal Ministry of Health, Abuja, Nigeria
7. WHO Nigeria Country Office, Abuja, Nigeria
8. International Health Consultancy, LLC, Atlanta, Georgia, USA
9. Department of Biostatistics, Epidemiology and Informatics, Perelman School of Medicine University of Pennsylvania Philadelphia, Pennsylvania, USA
10. Palliative and Advanced Illness Research (PAIR) Center, Perelman School of Medicine, University of Pennsylvania, Philadelphia, Pennsylvania, USA
11. College of Medicine, University of Ibadan, Ibadan, Nigeria

*****These authors contributed equally.

**Correspondence: Dr. Davies Adeloye,** Centre for Global Health, Usher Institute, University of Edinburgh, 30 West Richmond street, Edinburgh, EH8 9DX, UK. Email: [Davies.Adeloye@ed.ac.uk](mailto:Davies.Adeloye@ed.ac.uk)

**SUPPLEMENTARY MATERIAL**

**TABLE S1. Search terms on tobacco smoking in Nigeria.**

| **#** | **Searches** |
| --- | --- |
| 1 | africa/ or africa, sub-sahara/ or africa, western/ or nigeria/ |
| 2 | (incidence* or prevalence* or morbidity or mortality).tw. |
| 3 | (disease adj3 burden).tw. |
| 4 | 2 or 3 |
| 5 | exp smoking / or tobacco / or cigarettes |
| 6 | Exp NCDs risks/ |
| 7 | 5 or 6 |
| 8 | 1 and 5 and 7 |
| 9 | Limit 8 to “1990-current” |

**TABLE S2. Quality assessment of selected studies.**

| **Quality criteria** | **Assessment** | **Score** | **Maximum score** |
| --- | --- | --- | --- |
| Sampling method (was it representative of a target subnational population?) | Yes | 2 | 2 |
|  | Probably | 1 |  |
|  | No | 0 |  |
| Appropriateness of statistical analysis | Yes | 1 | 1 |
|  | No | 0 |  |
| Case ascertainment (was it based on accepted definition, other definitions, or not reported?) | Standard case definition | 2 | 2 |
|  | Other definitions | 1 |  |
|  | Not-reported | 0 |  |
| Total (*high (4-5), moderate (2-3), or low quality* (*0-1*)) | | | 5 |

**TABLE S3. Quality appraisal guide**

| Author | Sampling design | Statistical analysis | Case ascertainment | Total Score | Quality of evidence |
| --- | --- | --- | --- | --- | --- |
| Obaseki et al ^1^ | 1 | 1 | 1 | 3 | Moderate |
| Desalu et al ^2^ | 1 | 1 | 2 | 4 | High |
| Harris-Eze ^3^ | 1 | 1 | 1 | 3 | Moderate |
| Ozoh et al ^4^ | 1 | 1 | 1 | 3 | Moderate |
| Arute et al ^5^ | 1 | 1 | 1 | 3 | Moderate |
| Abiola et al ^6^ | 1 | 1 | 1 | 3 | Moderate |
| Adebiyi et al ^7^ | 1 | 1 | 2 | 4 | High |
| Adepoju et al ^8^ | 1 | 1 | 1 | 3 | Moderate |
| Agaba et al ^9^ | 1 | 1 | 2 | 4 | High |
| Agaku et al ^10^ | 1 | 1 | 2 | 4 | High |
| Aina et al ^11^ | 1 | 1 | 1 | 3 | Moderate |
| Azodo et al ^12^ | 1 | 1 | 2 | 3 | Moderate |
| Awopeju et al ^13^ | 1 | 1 | 2 | 3 | Moderate |
| Anyanwu et al ^14^ | 1 | 1 | 2 | 4 | High |
| Akinbodewa et al ^15^ | 1 | 1 | 2 | 4 | High |
| Babatunde et al ^16^ | 1 | 1 | 1 | 3 | Moderate |
| Babatunde et al ^17^ | 1 | 1 | 1 | 3 | Moderate |
| Dania et al ^18^ | 1 | 1 | 2 | 4 | High |
| Desalu et al ^19^ | 1 | 1 | 2 | 4 | High |
| Desalu et al ^20^ | 1 | 1 | 2 | 4 | High |
| Ebirim et al ^21^ | 1 | 1 | 2 | 4 | High |
| Ekanem et al ^22^ | 1 | 1 | 2 | 4 | High |
| Emerole et al ^23^ | 1 | 1 | 1 | 3 | Moderate |
| Fatoye & Morakinyo ^24^ | 1 | 1 | 1 | 3 | Moderate |
| Fawibe & Shittu ^25^ | 1 | 1 | 2 | 4 | High |
| Hussain et al ^26^ | 1 | 1 | 1 | 3 | Moderate |
| Ibekwe ^27^ | 1 | 1 | 1 | 3 | Moderate |
| Makanjuola et al ^28^ | 1 | 1 | 1 | 3 | Moderate |
| Morakinyo et al ^29^ | 1 | 1 | 2 | 3 | Moderate |
| Obot ^30^ | 1 | 1 | 1 | 3 | Moderate |
| Odey et al ^31^ | 1 | 1 | 1 | 3 | Moderate |
| Odeyemi et al ^32^ | 1 | 1 | 1 | 3 | Moderate |
| Odugbemi et al ^33^ | 1 | 1 | 1 | 3 | Moderate |
| Lawoyin et al ^34^ | 1 | 1 | 1 | 3 | Moderate |
| Ige et al ^35^ | 1 | 1 | 2 | 4 | High |
| Ugwuja et al ^36^ | 1 | 1 | 2 | 3 | Moderate |
| Odukoya et al ^37^ | 1 | 1 | 1 | 3 | Moderate |
| Okagua et al ^38^ | 1 | 1 | 2 | 4 | High |
| Oladapo et al ^39^ | 1 | 1 | 2 | 4 | High |
| Onofa et al ^40^ | 1 | 1 | 1 | 3 | Moderate |
| Onyeonoro et al ^41^ | 1 | 1 | 1 | 3 | Moderate |
| Oshodi et al ^42^ | 1 | 1 | 1 | 3 | Moderate |
| Owonaro & Eniojukan ^43^ | 1 | 1 | 2 | 4 | High |
| Owonaro & Eniojukan ^44^ | 1 | 1 | 1 | 3 | Moderate |
| Ozoh et al ^45^ | 1 | 1 | 1 | 3 | Moderate |
| Ozoh et al ^46^ | 1 | 1 | 1 | 3 | Moderate |
| Raji et al ^47^ | 1 | 1 | 2 | 4 | High |
| Raji et al ^48^ | 1 | 1 | 1 | 3 | Moderate |
| Salawu et al ^49^ | 1 | 1 | 1 | 3 | Moderate |
| Shehu & Idris ^50^ | 1 | 1 | 2 | 4 | High |
| Uwakwe & Gureje ^51^ | 1 | 1 | 1 | 3 | Moderate |
| Yisa et al ^52^ | 1 | 1 | 2 | 4 | High |
| Abasiubong et al ^53^ | 1 | 1 | 1 | 3 | Moderate |
| Gureje et al ^54^ | 1 | 1 | 2 | 4 | High |
| Lasebikan et al ^55^ | 1 | 1 | 1 | 3 | Moderate |
| Odenigbo et al ^56^ | 1 | 1 | 1 | 3 | Moderate |
| Forrest et al ^57^ | 1 | 1 | 2 | 4 | High |
| Oguoma et al ^58^ | 1 | 1 | 2 | 4 | High |
| Ezejimofor et al ^59^ | 1 | 1 | 2 | 4 | High |
| Ezekwesili et al ^60^ | 1 | 1 | 1 | 3 | Moderate |
| Ogah et al ^61^ | 1 | 1 | 2 | 4 | High |
| Suleiman et al ^62^ | 1 | 1 | 1 | 3 | Moderate |
| Ugwuja et al ^63^ | 1 | 1 | 2 | 4 | High |
| Wahab et al ^64^ | 1 | 1 | 2 | 4 | High |

**TABLE S4. All extracted data employed in analysis**

| Author | Study Period | Location | Geopolitical zone | Study design | Study Setting | Population xtics | Smoking type | Mean cigs per day | Mean pack years | Mean age | Median Age at initiation | Cases (all) | Sample (all) | Prev % (all) | Age (male) | Cases (male) | Sample (male) | Prev % (male) | Age (female) | Cases (female) | Sample (female) | Prev % (female) |
| --- | --- | --- | --- | --- | --- | --- | --- | --- | --- | --- | --- | --- | --- | --- | --- | --- | --- | --- | --- | --- | --- | --- |
| Obaseki et al | 2012 | Ile-Ife, Osun State | South-west | Population-based cross-sectional study | Rural | General population | Current |  |  | 53.5 |  | 26 | 1169 | 2.30 |  |  |  |  |  |  |  |  |
| Obaseki et al | 2012 | Ile-Ife, Osun State | South-west | Population-based cross-sectional study | Rural | General population | Ex |  |  | 53.5 |  | 97 | 1169 | 8.40 |  |  |  |  |  |  |  |  |
| Obaseki et al | 2012 | Ile-Ife, Osun State | South-west | Population-based cross-sectional study | Rural | General population | Ever |  |  | 53.5 |  | 123 | 1169 | 10.52 |  |  |  |  |  |  |  |  |
| Desalu et al | 2009 | Ido-Ekiti, Ekiti State | South-west | Population-based cross-sectional study | Rural | General population | Current |  |  | 55.5 |  | 10 | 385 | 2.60 |  |  |  |  |  |  |  |  |
| Desalu et al | 2009 | Ido-Ekiti, Ekiti State | South-west | Population-based cross-sectional study | Rural | General population | Ex |  |  | 55.5 |  | 36 | 391 | 9.20 |  |  |  |  |  |  |  |  |
| Desalu et al | 2009 | Ido-Ekiti, Ekiti State | South-west | Population-based cross-sectional study | Rural | General population | Ever |  |  | 55.5 |  | 46 | 385 | 11.96 |  |  |  |  |  |  |  |  |
| Harris-Eze | 1992 | Ibadan, Oyo State | South-west | Population-based cross-sectional study | Semi-urban | General population | Current |  |  | 35.8 |  | 128 | 805 | 15.90 |  |  |  |  |  |  |  |  |
| Harris-Eze | 1992 | Ibadan, Oyo State | South-west | Population-based cross-sectional study | Semi-urban | General population | Ex |  |  | 35.8 |  | 152 | 804 | 18.90 |  |  |  |  |  |  |  |  |
| Harris-Eze | 1992 | Ibadan, Oyo State | South-west | Population-based cross-sectional study | Semi-urban | General population | Ever |  |  | 35.8 |  | 280 | 805 | 34.78 |  |  |  |  |  |  |  |  |
| Ozoh et al | 2012 | Idi-Araba, Lagos State | South-west | Population-based cross-sectional study | Urban | General population | Current |  |  | 53.7 |  | 6 | 412 | 1.50 |  |  |  |  |  |  |  |  |
| Ozoh et al | 2012 | Idi-Araba, Lagos State | South-west | Population-based cross-sectional study | Urban | General population | Ex |  |  | 53.7 |  | 51 | 412 | 12.30 |  |  |  |  |  |  |  |  |
| Ozoh et al | 2012 | Idi-Araba, Lagos State | South-west | Population-based cross-sectional study | Urban | General population | Ever |  |  | 53.7 |  | 57 | 412 | 13.80 |  |  |  |  |  |  |  |  |
| Arute et al | 2015 | Abraka, Delta State | South-south | Population-based cross-sectional study | Semi-urban | Secondary school | Current | 2 |  | 17 | 13.5 | 14 | 400 | 3.50 |  |  |  |  |  |  |  |  |
| Arute et al | 2015 | Abraka, Delta State | South-south | Population-based cross-sectional study | Semi-urban | Secondary school | Ex |  |  | 17 |  | 14 | 400 | 3.50 |  |  |  |  |  |  |  |  |
| Arute et al | 2015 | Abraka, Delta State | South-south | Population-based cross-sectional study | Semi-urban | Secondary school | Ever |  |  | 17 |  | 28 | 400 | 7.00 | 17 | 20 | 208 | 9.62 | 17 | 8 | 192 | 4.17 |
| Abiola et al | 2014 | Mushin, Lagos State | South-west | Population-based cross-sectional study | Urban | Secondary school | Current |  |  | 16.4 | 12 | 59 | 402 | 14.70 | 16.4 | 56 | 255 | 22.00 | 16.4 | 3 | 147 | 2.00 |
| Adebiyi et al | 2009 | Kajola, Oyo State | South-west | Descriptive cross-sectional study | Rural | Secondary school | Current |  |  | 15 |  | 25 | 215 | 11.60 | 15 | 15 | 114 | 13.16 | 15 | 10 | 101 | 9.90 |
| Adebiyi et al | 2009 | Kajola, Oyo State | South-west | Descriptive cross-sectional study | Rural | Secondary school | Ex |  |  | 15 |  | 19 | 215 | 8.84 | 15 | 15 | 114 | 13.16 | 15 | 4 | 101 | 3.96 |
| Adebiyi et al | 2009 | Kajola, Oyo State | South-west | Descriptive cross-sectional study | Rural | Secondary school | Ever |  |  | 15 |  | 44 | 215 | 20.47 |  |  |  |  |  |  |  |  |
| Adepoju et al | 2011 | Osogbo, Osun State | South-west | Population-based cross-sectional study | Semi-urban | General population | Current | 22.9 | 9.16 | 42.1 | 18 | 66 | 759 | 8.70 | 42.1 | 48 | 364 | 13.20 | 42.1 | 18 | 395 | 4.60 |
| Adepoju et al | 2011 | Osogbo, Osun State | South-west | Population-based cross-sectional study | Semi-urban | General population | Ex |  |  | 42.1 |  | 101 | 759 | 13.30 | 42.1 | 69 | 364 | 18.90 | 42.1 | 32 | 395 | 8.10 |
| Adepoju et al | 2011 | Osogbo, Osun State | South-west | Population-based cross-sectional study | Semi-urban | General population | Ever |  |  | 42.1 |  | 167 | 759 | 22.00 | 42.1 | 117 | 364 | 32.14 | 42.1 | 50 | 395 | 12.66 |
| Agaba et al | 2014 | Jos, Plateau State | North-central | Descriptive cross-sectional study | Urban | Higher education institution | Current |  |  | 44 |  | 26 | 883 | 2.90 | 44 | 25 | 529 | 4.80 | 44 | 1 | 354 | 0.30 |
| Agaku et al | 2011 | Makurdi, Benue State | North-central | Population-based cross-sectional study | Urban | Secondary school | Current |  |  | 15 |  | 104 | 536 | 19.40 |  |  |  |  |  |  |  |  |
| Agaku et al | 2011 | Makurdi, Benue State | North-central | Population-based cross-sectional study | Urban | Secondary school | Ex |  |  | 15 |  | 43 | 536 | 8.00 |  |  |  |  |  |  |  |  |
| Agaku et al | 2011 | Makurdi, Benue State | North-central | Population-based cross-sectional study | Urban | Secondary school | Ever |  |  | 15 |  | 147 | 536 | 27.40 |  |  |  |  |  |  |  |  |
| Aina et al | 2007 | Idi-Aaraba, Lagos State | South-west | Descriptive cross-sectional study | Urban | Health professional students | Current |  |  | 23.5 |  | 17 | 408 | 3.93 | 23.5 | 15 | 203 | 7.39 | 23.5 | 2 | 230 | 0.87 |
| Aina et al | 2007 | Idi-Aaraba, Lagos State | South-west | Descriptive cross-sectional study | Urban | Health professional students | Ex |  |  | 23.5 |  | 8 | 408 | 1.85 | 23.5 | 8 | 203 | 3.94 | 23.5 |  |  |  |
| Aina et al | 2007 | Idi-Aaraba, Lagos State | South-west | Descriptive cross-sectional study | Urban | Health professional students | Ever |  |  | 23.5 |  | 25 | 408 | 6.13 | 23.5 | 23 | 203 | 11.33 | 23.5 | 2 | 230 | 0.87 |
| Azodo et al | 2011 | Abuja, Nassarawa & Kano | North-central, North-west | Descriptive cross-sectional study | Urban | Prison workers | Current | 5 |  | 32.5 |  | 39 | 146 | 26.71 | 32.5 | 38 | 97 | 39.20 | 32.5 | 1 | 49 | 2.04 |
| Awopeju et al | 2012 | Ile-Ife, Osun State and Idi-Araba, Lagos State | South-west | Descriptive cross-sectional study | Mixed | Health professional students | Current |  |  | 32.5 | 13 | 34 | 675 | 5.04 | 32.5 | 23 | 330 | 3.40 | 32.5 | 11 | 345 | 1.60 |
| Awopeju et al | 2012 | Ile-Ife, Osun State and Idi-Araba, Lagos State | South-west | Descriptive cross-sectional study | Mixed | Health professional students | Ex |  |  | 32.5 |  | 87 | 675 | 12.86 | 32.5 | 56 | 330 | 20.50 | 32.5 | 31 | 345 | 10.30 |
| Awopeju et al | 2012 | Ile-Ife, Osun State and Idi-Araba, Lagos State | South-west | Descriptive cross-sectional study | Mixed | Health professional students | Ever |  |  | 32.5 |  | 121 | 675 | 17.90 | 32.5 | 79 | 330 | 23.90 | 32.5 | 42 | 345 | 11.90 |
| Anyanwu et al | 2015 | Abakaliki, Ebonyi State | South-east | Population-based cross-sectional study | Urban | Secondary school | Current |  |  | 17 |  | 89 | 620 | 14.40 | 17 | 47 | 297 | 15.82 | 17 | 42 | 323 | 13.00 |
| Akinbodewa et al | 2014 | Akure & Ondo, Ondo State | South-west | Descriptive cross-sectional study | Mixed | General population | Ever |  |  | 44.7 |  | 38 | 1183 | 4.50 |  |  |  |  |  |  |  |  |
| Babatunde et al | 2016 | Ilorin, Kwara State | North-central | Population-based cross-sectional study | Urban | Secondary school | Current |  |  | 15 |  | 272 | 2000 | 13.60 | 15 | 160 | 1100 | 14.50 | 15 | 103 | 900 | 11.40 |
| Babatunde et al | 2011 | Ido-Ekiti, Ekiti State | South-west | Descriptive cross-sectional study | Semi-urban | Higher education institution | Current |  |  | 22 |  | 41 | 300 | 13.70 |  |  |  |  |  |  |  |  |
| Babatunde et al | 2011 | Ido-Ekiti, Ekiti State | South-west | Descriptive cross-sectional study | Semi-urban | Higher education institution | Ex |  |  | 22 |  | 25 | 300 | 8.30 |  |  |  |  |  |  |  |  |
| Babatunde et al | 2011 | Ido-Ekiti, Ekiti State | South-west | Descriptive cross-sectional study | Semi-urban | Higher education institution | Ever |  |  | 22 |  | 66 | 300 | 22.00 |  |  |  |  |  |  |  |  |
| Dania et al | 2015 | Yaba, Lagos State | South-west | Descriptive cross-sectional study | Urban | Health professional students | Current |  |  | 21.4 | 18 | 3 | 250 | 1.20 | 21.4 | 2 | 133 | 1.50 | 21.4 | 1 | 117 | 0.85 |
| Dania et al | 2015 | Yaba, Lagos State | South-west | Descriptive cross-sectional study | Urban | Health professional students | Ex |  |  | 21.4 |  | 21 | 250 | 8.40 | 21.4 | 14 | 133 | 10.53 | 21.4 | 7 | 117 | 5.98 |
| Dania et al | 2015 | Yaba, Lagos State | South-west | Descriptive cross-sectional study | Urban | Health professional students | Ever |  |  | 21.4 |  | 24 | 250 | 9.60 | 21.4 | 16 | 133 | 12.03 | 21.4 | 8 | 117 | 6.84 |
| Desalu et al | 2007 | Yola, Adamawa State | North-east | Population-based cross-sectional study | Semi-urban | General population | Current | 10 |  | 35.8 | 18.6 | 572 | 1793 | 31.90 | 35.8 | 408 | 901 | 45.30 | 35.8 | 164 | 892 | 18.40 |
| Desalu et al | 2007 | Yola, Adamawa State | North-east | Population-based cross-sectional study | Semi-urban | General population | Ex |  |  | 35.8 |  | 107 | 1793 | 6.00 | 35.8 | 82 | 901 | 9.08 | 35.8 | 25 | 892 | 2.79 |
| Desalu et al | 2007 | Yola, Adamawa State | North-east | Population-based cross-sectional study | Semi-urban | General population | Ever |  |  | 35.8 |  | 679 | 1793 | 37.90 | 35.8 | 490 | 901 | 54.38 | 35.8 | 189 | 892 | 21.19 |
| Desalu et al (cough) | 2009 | Ilorin, Kwara State | North-central | Population-based cross-sectional study | Urban | General population | Current |  |  | 40 |  | 38 | 472 | 8.10 |  |  |  |  |  |  |  |  |
| Desalu et al (cough) | 2009 | Ilorin, Kwara State | North-central | Population-based cross-sectional study | Urban | General population | Ex |  |  | 40 |  | 21 | 472 | 4.40 |  |  |  |  |  |  |  |  |
| Desalu et al (cough) | 2009 | Ilorin, Kwara State | North-central | Population-based cross-sectional study | Urban | General population | Ever |  |  | 40 |  | 59 | 472 | 12.50 |  |  |  |  |  |  |  |  |
| Ebirim et al | 2013 | Owerri, Imo State | South-east | Descriptive cross-sectional study | Urban | Secondary school | Current | 7.5 |  | 16 | 14 | 106 | 944 | 11.20 |  |  |  |  |  |  |  |  |
| Ebirim et al | 2013 | Owerri, Imo State | South-east | Descriptive cross-sectional study | Urban | Secondary school | Ex |  |  | 16 |  | 38 | 944 | 4.10 |  |  |  |  |  |  |  |  |
| Ebirim et al | 2013 | Owerri, Imo State | South-east | Descriptive cross-sectional study | Urban | Secondary school | Ever |  |  | 16 |  | 144 | 944 | 15.30 |  |  |  |  |  |  |  |  |
| Ekanem et al | 2008 | Abuja, FCT | North-central | Population-based cross-sectional study | Urban | Secondary school | Current |  |  | 15 |  | 49 | 1399 | 3.50 | 15 | 43 | 769 | 5.60 | 15 | 8 | 630 | 1.30 |
| Ekanem et al | 2008 | Abuja, FCT | North-central | Population-based cross-sectional study | Urban | Secondary school | Ex |  |  | 15 |  | 119 | 1399 | 8.50 |  |  |  |  |  |  |  |  |
| Ekanem et al | 2008 | Abuja, FCT | North-central | Population-based cross-sectional study | Urban | Secondary school | Ever |  |  | 15 |  | 168 | 1399 | 12.00 | 15 | 116 | 769 | 15.10 | 15 | 50 | 630 | 8.00 |
| Ekanem et al | 2008 | Ibadan, Oyo State | South-west | Population-based cross-sectional study | Urban | Secondary school | Current |  |  | 15 |  | 22 | 637 | 3.50 | 15 | 5 | 350 | 1.40 | 15 | 16 | 287 | 5.50 |
| Ekanem et al | 2008 | Ibadan, Oyo State | South-west | Population-based cross-sectional study | Urban | Secondary school | Ex |  |  | 15 |  | 8 | 637 | 1.20 |  |  |  |  |  |  |  |  |
| Ekanem et al | 2008 | Ibadan, Oyo State | South-west | Population-based cross-sectional study | Urban | Secondary school | Ever |  |  | 15 |  | 30 | 637 | 4.70 | 15 | 11 | 350 | 3.00 | 15 | 18 | 287 | 6.40 |
| Ekanem et al | 2008 | Lagos State | South-west | Population-based cross-sectional study | Urban | Secondary school | Current |  |  | 15 |  | 38 | 1461 | 2.60 | 15 | 22 | 804 | 2.80 | 15 | 12 | 657 | 1.80 |
| Ekanem et al | 2008 | Lagos State | South-west | Population-based cross-sectional study | Urban | Secondary school | Ex |  |  | 15 |  | 75 | 1461 | 5.10 |  |  |  |  |  |  |  |  |
| Ekanem et al | 2008 | Lagos State | South-west | Population-based cross-sectional study | Urban | Secondary school | Ever |  |  | 15 |  | 112 | 1461 | 7.70 | 15 | 73 | 804 | 9.10 | 15 | 36 | 657 | 5.50 |
| Ekanem et al | 2008 | Kano State | North-west | Population-based cross-sectional study | Urban | Secondary school | Current |  |  | 15 |  | 59 | 944 | 6.20 | 15 | 27 | 236 | 11.40 | 15 | 39 | 708 | 5.50 |
| Ekanem et al | 2008 | Kano State | North-west | Population-based cross-sectional study | Urban | Secondary school | Ex |  |  | 15 |  | 93 | 944 | 9.90 |  |  |  |  |  |  |  |  |
| Ekanem et al | 2008 | Kano State | North-west | Population-based cross-sectional study | Urban | Secondary school | Ever |  |  | 15 |  | 152 | 944 | 16.10 | 15 | 152 | 519 | 29.20 | 15 | 16 | 425 | 3.70 |
| Ekanem et al | 2008 | Cross River State | South-south | Population-based cross-sectional study | Urban | Secondary school | Current |  |  | 15 |  | 42 | 1018 | 4.10 | 15 | 38 | 560 | 6.80 | 15 | 5 | 458 | 1.20 |
| Ekanem et al | 2008 | Cross River State | South-south | Population-based cross-sectional study | Urban | Secondary school | Ex |  |  | 15 |  | 95 | 1018 | 9.30 |  |  |  |  |  |  |  |  |
| Ekanem et al | 2008 | Cross River State | South-south | Population-based cross-sectional study | Urban | Secondary school | Ever |  |  | 15 |  | 136 | 1018 | 13.40 | 15 | 78 | 560 | 13.90 | 15 | 45 | 458 | 9.90 |
| Emerole et al | 2007 | Owerri, Imo State | South-east | Descriptive cross-sectional study | Urban | Higher education institution | Current |  |  | 53 |  | 9 | 241 | 3.73 |  |  |  |  |  |  |  |  |
| Fatoye & Morakinyo | 2001 | Ilesa, Osun State | South-west | Descriptive cross-sectional study | Mixed | Secondary school | Current |  |  | 17 |  | 17 | 567 | 3.00 |  |  |  |  |  |  |  |  |
| Fawibe & Shittu | 2009 | Ilorin, Kwara State | North-central | Descriptive cross-sectional study | Urban | Higher education institution | Current | 5.2 |  | 21.6 | 15.5 | 100 | 1754 | 5.70 | 21.6 | 88 | 1148 | 7.70 | 21.6 | 12 | 606 | 2.00 |
| Fawibe & Shittu | 2009 | Ilorin, Kwara State | North-central | Descriptive cross-sectional study | Urban | Higher education institution | Ex |  |  | 21.6 |  | 200 | 1754 | 11.40 | 21.6 | 174 | 1148 | 15.20 | 21.6 | 25 | 606 | 4.20 |
| Fawibe & Shittu | 2009 | Ilorin, Kwara State | North-central | Descriptive cross-sectional study | Urban | Higher education institution | Ever |  |  | 21.6 |  | 300 | 1754 | 17.10 | 21.6 | 263 | 1148 | 22.90 | 21.6 | 38 | 606 | 6.20 |
| Hussain et al | 2007 | Lagos State | South-west | Descriptive cross-sectional study | Urban | Soldiers | Current |  |  | 31.5 |  | 173 | 853 | 20.30 |  |  |  |  |  |  |  |  |
| Ibekwe | 2012 | Oghara, Delta State | South-south | Descriptive cross-sectional study | Rural | General population | Current |  |  | 36.7 |  | 43 | 272 | 15.80 |  |  |  |  |  |  |  |  |
| Makanjuola et al | 2004 | Ilorin, Kwara State | North-central | Descriptive cross-sectional study | Urban | Health professional students | Current |  |  | 22.4 |  | 29 | 961 | 3.20 |  |  |  |  |  |  |  |  |
| Makanjuola et al | 2004 | Ilorin, Kwara State | North-central | Descriptive cross-sectional study | Urban | Health professional students | Ex |  |  | 22.4 |  | 66 | 961 | 7.30 |  |  |  |  |  |  |  |  |
| Makanjuola et al | 2004 | Ilorin, Kwara State | North-central | Descriptive cross-sectional study | Urban | Health professional students | Ever |  |  | 22.4 |  | 95 | 961 | 10.50 |  |  |  |  |  |  |  |  |
| Morakinyo et al | 2003 | Ibadan, Oyo State | South-west | Population-based cross-sectional study | Urban | Street adolescents | Current |  |  | 15 | 12 | 18 | 180 | 10.00 |  |  |  |  |  |  |  |  |
| Morakinyo et al | 2003 | Ibadan, Oyo State | South-west | Population-based cross-sectional study | Urban | Street adolescents | Ex |  |  | 15 |  | 8 | 180 | 4.40 |  |  |  |  |  |  |  |  |
| Morakinyo et al | 2003 | Ibadan, Oyo State | South-west | Population-based cross-sectional study | Urban | Street adolescents | Ever |  |  | 15 |  | 26 | 180 | 14.40 |  |  |  |  |  |  |  |  |
| Obot | 1990 | Jos, Plateau State | North-central | Population-based cross-sectional study | Mixed | General population | Current | 10 |  | 41 |  | 341 | 1271 | 26.80 |  |  |  |  |  |  |  |  |
| Obot | 1990 | Jos, Plateau State | North-central | Population-based cross-sectional study | Mixed | General population | Ex |  |  | 41 |  | 60 | 1271 | 4.70 |  |  |  |  |  |  |  |  |
| Obot | 1990 | Jos, Plateau State | North-central | Population-based cross-sectional study | Mixed | General population | Ever |  |  | 41 |  | 400 | 1271 | 31.50 | 41 | 388 | 1137 | 34.10 | 41 | 13 | 134 | 9.70 |
| Odey et al | 2012 | Calabar, Cross River State | South-south | Descriptive cross-sectional study | Urban | Secondary school | Current |  |  | 15 |  | 24 | 375 | 6.40 | 15 | 19 | 146 | 13.00 | 15 | 5 | 229 | 2.10 |
| Odeyemi et al | 2009 | National | National | Descriptive cross-sectional study | Mixed | Secondary school | Current |  |  | 16 |  | 202 | 1183 | 17.10 |  |  |  |  |  |  |  |  |
| Odeyemi et al | 2009 | National | National | Descriptive cross-sectional study | Mixed | Secondary school | Ex |  |  | 16 |  | 110 | 1183 | 9.30 |  |  |  |  |  |  |  |  |
| Odeyemi et al | 2009 | National | National | Descriptive cross-sectional study | Mixed | Secondary school | Ever |  |  | 16 |  | 312 | 1183 | 26.40 |  |  |  |  |  |  |  |  |
| Odugbemi et al | 2010 | Tejuosho, Lagos | South-west | Descriptive cross-sectional study | Urban | Traders | Current |  |  | 43.3 |  | 18 | 400 | 4.50 | 45.48 | 17 | 103 | 16.50 | 42.29 | 1 | 297 | 0.34 |
| Odugbemi et al | 2010 | Tejuosho, Lagos | South-west | Descriptive cross-sectional study | Urban | Traders | Ex |  |  | 43.3 |  | 11 | 400 | 2.70 | 43.3 | 10 | 103 | 9.70 | 43.3 | 1 | 297 | 0.34 |
| Odugbemi et al | 2010 | Tejuosho, Lagos | South-west | Descriptive cross-sectional study | Urban | Traders | Ever |  |  | 43.3 |  | 29 | 400 | 7.20 | 43.3 | 27 | 103 | 26.20 | 43.3 | 2 | 297 | 0.68 |
| Lawoyin et al | 1998 | Idikan Ibadan, Oyo State | South-west | Population-based cross-sectional study | Rural | General population | Ever |  |  | 55 |  | 397 | 2144 | 18.50 | 55 | 165 | 892 | 18.50 | 55 | 232 | 1252 | 18.50 |
| Ige et al | 2013 | Ibadan, Oyo State | South-west | Descriptive cross-sectional study | Urban | Higher education institution | Current |  |  | 37.4 |  | 10 | 525 | 1.90 | 37.4 | 9 | 269 | 3.30 | 37.4 | 1 | 256 | 0.40 |
| Ugwuja et al | 2008 | Abakaliki, Ebonyi State | South-east | Descriptive cross-sectional study | Urban | Civil servants | Current |  |  | 40.9 |  | 12 | 205 | 5.90 | 40.9 |  | 106 |  | 40.9 |  | 99 |  |
| Odukoya et al | 2011 | Lagos State | South-west | Descriptive cross-sectional study | Urban | Secondary school | Current |  |  | 15 |  | 95 | 989 | 9.60 | 15 | 66 | 523 | 12.70 | 15 | 29 | 466 | 6.30 |
| Okagua et al | 2015 | Port-Harcourt, Rivers State | South-south | Descriptive cross-sectional study | Urban | Secondary school | Current |  |  | 15 |  | 39 | 1120 | 3.30 |  |  |  |  |  |  |  |  |
| Okagua et al | 2015 | Port-Harcourt, Rivers State | South-south | Descriptive cross-sectional study | Urban | Secondary school | Ex |  |  | 15 |  | 41 | 1120 | 3.80 | 15 |  |  | 9.70 | 15 |  |  | 4.00 |
| Okagua et al | 2015 | Port-Harcourt, Rivers State | South-south | Descriptive cross-sectional study | Urban | Secondary school | Ever |  |  | 15 |  | 80 | 1120 | 7.10 |  |  |  |  |  |  |  |  |
| Oladapo et al | 2015 | Egbeda, Oyo State | South-west | Descriptive cross-sectional study | Rural | General population | Current |  |  | 42.1 |  | 33 | 2000 | 1.70 | 42.1 | 32 | 873 | 3.80 | 42.1 | 1 | 1127 | 0.10 |
| Onofa et al | 2016 | Abeokuta, Ogun State | South-west | Descriptive cross-sectional study | Urban | Higher education institution | Ever |  |  | 23.6 | 13.6 | 177 | 1233 | 14.40 | 23.6 |  | 691 |  | 23.6 |  | 542 |  |
| Onyeonoro et al | 2015 | Umuahia, Abia State | South-east | Population-based cross-sectional study | Semi-urban | General population | Current |  |  | 40.5 |  | 385 | 2983 | 13.00 | 40.5 |  | 1430 |  | 40.5 |  | 1553 |  |
| Oshodi et al | 2008 | Surulere, Lagos State | South-west | Descriptive cross-sectional study | Urban | Secondary school | Current |  |  | 15.9 |  | 11 | 366 | 3.00 |  |  |  |  |  |  |  |  |
| Oshodi et al | 2008 | Surulere, Lagos State | South-west | Descriptive cross-sectional study | Urban | Secondary school | Ex |  |  | 15.9 |  | 8 | 366 | 2.20 |  |  |  |  |  |  |  |  |
| Oshodi et al | 2008 | Surulere, Lagos State | South-west | Descriptive cross-sectional study | Urban | Secondary school | Ever |  |  | 15.9 |  | 19 | 366 | 5.20 | 15.9 | 12 | 175 | 6.90 | 15.9 | 7 | 227 | 3.10 |
| Owonaro & Eniojukan | 2015 | Amassoma, Bayelsa State | South-south | Descriptive cross-sectional study | Urban | General population | Current |  |  | 24 | 20.5 | 141 | 254 | 55.50 | 24 |  | 187 |  | 24 |  | 67 |  |
| Owonaro & Eniojukan | 2015 | Amassoma, Bayelsa State | South-south | Descriptive cross-sectional study | Urban | General population | Ex |  |  | 24 |  | 23 | 254 | 9.10 |  |  |  |  |  |  |  |  |
| Owonaro & Eniojukan | 2015 | Amassoma, Bayelsa State | South-south | Descriptive cross-sectional study | Urban | General population | Ever |  |  | 24 |  | 164 | 254 | 64.60 |  |  |  |  |  |  |  |  |
| Owonaro & Eniojukan | 2015 | Amassoma, Bayelsa State | South-south | Descriptive cross-sectional study | Urban | Higher education institution | Current | 5 |  | 24 | 18 | 26 | 201 | 12.90 | 24 | 19 | 107 | 17.80 | 24 | 7 | 94 | 7.80 |
| Owonaro & Eniojukan | 2015 | Opokuma, Bayelsa State | South-south | Descriptive cross-sectional study | Rural | General population | Current |  |  | 30 | 18 | 27 | 252 | 10.71 | 30 |  | 156 |  | 30 |  | 96 |  |
| Owonaro & Eniojukan | 2015 | Opokuma, Bayelsa State | South-south | Descriptive cross-sectional study | Rural | General population | Ex |  |  | 30 |  | 24 | 252 | 9.52 |  |  |  |  |  |  |  |  |
| Owonaro & Eniojukan | 2015 | Opokuma, Bayelsa State | South-south | Descriptive cross-sectional study | Rural | General population | Ever |  |  | 30 |  | 51 | 252 | 20.24 |  |  |  |  |  |  |  |  |
| Ozoh et al | 2014 | Lagos mainland, Lagos State | South-west | Descriptive cross-sectional study | Urban | Commercial drivers | Current |  |  | 42.36 |  | 60 | 500 | 32.00 |  |  |  |  |  |  |  |  |
| Ozoh et al | 2014 | Lagos mainland, Lagos State | South-west | Descriptive cross-sectional study | Urban | Commercial drivers | Ex |  |  | 42.36 |  | 226 | 500 | 25.20 |  |  |  |  |  |  |  |  |
| Ozoh et al | 2014 | Lagos mainland, Lagos State | South-west | Descriptive cross-sectional study | Urban | Commercial drivers | Ever |  |  | 42.36 |  | 286 | 500 | 57.20 |  |  |  |  |  |  |  |  |
| Ozoh et al | 2017 | Lagos mainland, Lagos State | South-west | Descriptive cross-sectional study | Urban | Commercial drivers | Current |  | 7.6 | 44 | 21.9 | 123 | 414 | 29.70 |  |  |  |  |  |  |  |  |
| Ozoh et al | 2017 | Lagos mainland, Lagos State | South-west | Descriptive cross-sectional study | Urban | Commercial drivers | Ex |  |  | 44 |  | 46 | 414 | 11.10 |  |  |  |  |  |  |  |  |
| Ozoh et al | 2017 | Lagos mainland, Lagos State | South-west | Descriptive cross-sectional study | Urban | Commercial drivers | Ever |  |  | 44 |  | 169 | 414 | 40.80 |  |  |  |  |  |  |  |  |
| Raji et al | 2012 | Sokoto, Sokoto State | North-west | Descriptive cross-sectional study | Urban | Secondary school | Current |  |  | 16.6 |  | 19 | 228 | 8.30 | 16.6 | 15 | 181 | 8.29 | 16.6 | 4 | 47 | 8.51 |
| Raji et al | 2017 | Sokoto, Sokoto State | North-west | Descriptive cross-sectional study | Urban | Street adolescents | Current |  |  | 15 |  | 7 | 213 | 3.30 | 15 |  | 188 |  | 15 |  | 25 |  |
| Raji et al | 2017 | Sokoto, Sokoto State | North-west | Descriptive cross-sectional study | Urban | Street adolescents | Ex |  |  | 15 |  | 17 | 213 | 8.00 |  |  |  |  |  |  |  |  |
| Raji et al | 2017 | Sokoto, Sokoto State | North-west | Descriptive cross-sectional study | Urban | Street adolescents | Ever |  |  | 15 |  | 24 | 213 | 11.30 |  |  |  |  |  |  |  |  |
| Salawu et al | 2009 | Yola, Adamawa | North-east | Population-based cross-sectional study | Semi-urban | Street adolescents | Current |  |  | 15 |  | 58 | 171 | 33.92 | 15 | 44 | 109 | 40.37 | 15 | 14 | 62 | 22.58 |
| Salawu et al | 2009 | Yola, Adamawa | North-east | Population-based cross-sectional study | Semi-urban | Street adolescents | Ex |  |  | 15 |  | 28 | 171 | 16.37 | 15 | 18 | 109 | 16.51 | 15 | 10 | 62 | 16.13 |
| Salawu et al | 2009 | Yola, Adamawa | North-east | Population-based cross-sectional study | Semi-urban | Street adolescents | Ever |  |  | 15 |  | 86 | 171 | 50.29 | 15 | 62 | 109 | 56.88 | 15 | 24 | 62 | 38.71 |
| Shehu & Idris | 2004 | Saria, Kaduna State | North-west | Descriptive cross-sectional study | Semi-urban | Secondary school | Ever |  |  | 15 |  | 33 | 350 | 9.40 | 15 |  | 262 |  | 15 |  | 88 |  |
| Uwakwe & Gureje | 2016 | National | National | Population-based cross-sectional study | Mixed | General population | Current |  |  | 41 |  | 282 | 6752 | 4.18 | 41 | 269 | 3427 | 24.90 | 41 | 13 | 3315 | 0.40 |
| Uwakwe & Gureje | 2016 | National | National | Population-based cross-sectional study | Mixed | General population | Ex |  |  | 41 |  | 855 | 6752 | 12.82 | 41 | 811 | 3427 | 7.70 | 41 | 44 | 3315 | 1.30 |
| Uwakwe & Gureje | 2016 | National | National | Population-based cross-sectional study | Mixed | General population | Ever |  |  | 41 |  | 1137 | 6752 | 17.00 | 41 | 1080 | 3427 | 32.60 | 41 | 57 | 3315 | 1.70 |
| Yisa et al | 2009 | Ibadan, Oyo State | South-west | Descriptive cross-sectional study | Urban | Secondary school | Ever |  |  | 15 |  | 11 | 510 | 2.08 |  |  |  |  |  |  |  |  |
| Abasiubong et al | 2005 | Eket, Akwa-Ibom State | South-south | Descriptive cross-sectional study | Mixed | Secondary school | Current |  |  | 17.1 |  | 47 | 254 | 34.80 |  |  |  |  |  |  |  |  |
| Gureje et al | 2007 | National | National | Population-based cross-sectional study | Mixed | General population | Current |  |  | 34 |  | 230 | 6752 | 3.40 |  |  |  |  |  |  |  |  |
| Gureje et al | 2007 | National | National | Population-based cross-sectional study | Mixed | General population | Ex |  |  | 34 |  | 905 | 6752 | 13.40 |  |  |  |  |  |  |  |  |
| Gureje et al | 2007 | National | National | Population-based cross-sectional study | Mixed | General population | Ever |  |  | 34 |  | 1134 | 6752 | 16.80 | 34 | 1085 | 3307 | 32.80 | 34 | 52 | 3445 | 1.50 |
| Lasebikan et al | 2016 | Oyo State | South-west | Population-based cross-sectional study | Rural | General population | Current | 23.7 |  | 44.5 |  | 248 | 1203 | 20.60 | 44.5 | 181 | 623 | 29.10 | 44.5 | 67 | 580 | 11.60 |
| Lasebikan et al | 2016 | Oyo State | South-west | Population-based cross-sectional study | Rural | General population | Ex |  |  | 44.5 |  | 158 | 1203 | 13.10 |  |  |  |  |  |  |  |  |
| Lasebikan et al | 2016 | Oyo State | South-west | Population-based cross-sectional study | Rural | General population | Ever |  |  | 44.5 |  | 406 | 1203 | 33.70 |  |  |  |  |  |  |  |  |
| Odenigbo et al | 2008 | Asaba, Delta State | South-south | Population-based cross-sectional study | Semi-urban | General population | Current |  |  | 41.59 |  | 2 | 100 | 2.00 |  |  |  |  |  |  |  |  |
| Forrest et al | 1992 | Benin, Edo State | South-south | Population-based cross-sectional study | Urban | Civil servants | Ever |  |  | 41.1 |  | 53 | 464 | 11.45 | 41.1 | 52 | 286 | 18.20 | 41.1 | 1 | 178 | 0.60 |
| Forrest et al | 1992 | Benin, Edo State | South-south | Population-based cross-sectional study | Urban | Civil servants | Ever |  |  | 43.8 |  | 24 | 335 | 7.13 | 43.8 | 23 | 212 | 10.80 | 43.8 | 1 | 123 | 0.80 |
| Oguoma et al | 2015 | Kwale, Delta State | South-south | Population-based cross-sectional study | Mixed | General population | Current |  |  | 39.9 |  | 14 | 422 | 3.40 | 39.9 | 10 | 145 | 7.00 | 39.9 | 4 | 277 | 1.50 |
| Oguoma et al | 2015 | Kwale, Delta State | South-south | Population-based cross-sectional study | Mixed | General population | Ex |  |  | 39.9 |  | 31 | 422 | 7.80 | 39.9 | 29 | 145 | 21.30 | 39.9 | 2 | 277 | 0.80 |
| Oguoma et al | 2015 | Kwale, Delta State | South-south | Population-based cross-sectional study | Mixed | General population | Ever |  |  | 39.9 |  | 45 | 422 | 11.20 | 39.9 | 39 | 145 | 28.30 | 39.9 | 6 | 277 | 2.30 |
| Ezejimofor et al | 2014 | Niger Delta, Delta State | South-south | Community-based cross-sectional study | Rural | General population | Ever |  |  | 44.32 |  | 339 | 2028 | 16.72 | 44.32 |  | 871 |  | 44.32 |  | 1157 | 249.00 |
| Ezekwesili et al | 2016 | Anambra State | South-east | Population-based cross-sectional study | Mixed | General population | Current |  |  | 38 |  | 28 | 912 | 3.07 |  |  |  |  |  |  |  |  |
| Ogah et al. 2013 | 2012 | Umuahia, Abia State | South-east | Population-based cross-sectional study | Mixed | General population | Ever |  |  | 41.7 |  | 398 | 2983 | 13.30 | 41.5 | 383 | 1430 | 26.80 | 41.8 | 15 | 1553 | 1.00 |
| Suleiman et al. 2013 | 2011 | Amassoma, Bayelsa State | South-south | Descriptive cross-sectional study | Semi-urban | General population | Ever |  |  | 50.5 |  | 57 | 400 | 14.30 |  |  |  |  |  |  |  |  |
| Ugwuuja et al | 2015 | Igbeagu, Ebonyi State | South-east | Population-based cross-sectional study | Rural | Agrarian community | Ever |  |  | 41 |  | 8 | 267 | 3.00 |  |  |  |  |  |  |  |  |
| Wahab et al | 2006 | Katsina, Katsina State | North-west | Population-based cross-sectional study | Urban | General population | Current |  |  | 37.6 |  | 14 | 300 | 4.70 | 38 | 11 | 129 | 8.50 | 37.2 | 3 | 171 | 1.80 |

**FIGURE S1. Crude prevalence rate of current smokers in Nigeria, by geopolitical zones.**

**FIGURE S2. Crude prevalence rate of ever smokers in Nigeria, by geopolitical zones.**

**FIGURE S3. Pooled mean cigarettes consumed per person per day in Nigeria.**

**FIGURE S4. Meta-regression modelling**

**Current smokers**

**Ever smokers**

**REFERENCES**

1. Obaseki D, Erhabor G, Burney P, Buist S, Awopeju O, Gnatiuc L. The prevalence of COPD in an African city: Results of the BOLD study, Ile-Ife, Nigeria. In: Eur Respiratory Soc; 2013.

2. Desalu OO. Prevalence of chronic bronchitis and tobacco smoking in some rural communities in Ekiti state, Nigeria. *The Nigerian postgraduate medical journal.* 2011;18(2):91-97.

3. Harris-Eze AO. Smoking habits and chronic bronchitis in Nigerian soldiers. *East African medical journal.* 1993;70(12):763-767.

4. Ozoh O, Balogun B, Oguntunde O, et al. The prevalence and determinants of COPD in an urban community in Lagos, Nigeria. In: Eur Respiratory Soc; 2013.

5. Arute J, Oyita G, Eniojukan J. Substance Abuse among Adolescents: 2. Prevalence and Patterns of Cigarette smoking among senior secondary school students in Abraka, Delta State, Nigeria. *IOSR Journal of Pharmacy.* 2015;5(1):40-47.

6. Abiola A, Balogun O, Odukoya O, et al. Age of initiation, Determinants and Prevalence of Cigarette Smoking among Teenagers in Mushin Local Government Area of Lagos State, Nigeria. *Asian Pacific journal of cancer prevention : APJCP.* 2016;17(3):1209-1214.

7. Adebiyi AO, Faseru B, Sangowawa AO, Owoaje ET. Tobacco use amongst out of school adolescents in a Local Government Area in Nigeria. *Substance abuse treatment, prevention, and policy.* 2010;5:24.

8. Adepoju EG, Olowookere SA, Adeleke NA, Afolabi OT, Olajide FO, Aluko OO. A population based study on the prevalence of cigarette smoking and smokers' characteristics at osogbo, Nigeria. *Tobacco use insights.* 2013;6:1-5.

9. Agaba EI, Akanbi MO, Agaba PA, et al. A survey of non-communicable diseases and their risk factors among university employees: a single institutional study. *Cardiovascular journal of Africa.* 2017;28(6):377-384.

10. Agaku IT, Filippidis FT. Prevalence, determinants and impact of unawareness about the health consequences of tobacco use among 17,929 school personnel in 29 African countries. *BMJ open.* 2014;4(8):e005837.

11. Aina BA, Oyerinde OO, Joda AE, Dada OO. Cigarette smoking among healthcare professional students of University of Lagos and Lagos University Teaching Hospital (LUTH), Idi-Araba, Lagos, Nigeria. *Nigerian quarterly journal of hospital medicine.* 2009;19(1):42-46.

12. Azodo CC, Omili M. Tobacco use, Alcohol Consumption and Self-rated Oral Health among Nigerian Prison Officials. *International journal of preventive medicine.* 2014;5(11):1364-1371.

13. Awopeju O, Erhabor G, Awosusi B, Awopeju O, Adewole O, Irabor I. Smoking prevalence and attitudes regarding its control among health professional students in South-Western Nigeria. *Annals of medical and health sciences research.* 2013;3(3):355-360.

14. Anyanwu OU, Ibekwe RC, Ojinnaka NC. Pattern of substance abuse among adolescent secondary school students in Abakaliki. *Cogent Medicine.* 2016;3(1):1272160.

15. Akinbodewa AA, Adejumo AO, Koledoye OV, et al. Community screening for pre-hypertension, traditional risk factors and markers of chronic kidney disease in Ondo State, South-Western Nigeria. *The Nigerian postgraduate medical journal.* 2017;24(1):25-30.

16. Babatunde LS, Babatunde OT, Oladeji SM, Ashipa T. Prevalence and determinants of susceptibility to cigarette smoking among non-smoking senior secondary school students in Ilorin, North Central Nigeria. *International journal of adolescent medicine and health.* 2017.

17. Babatunde OA, Elegbede OE, Ayodele LM, Atoyebi OA, Ibirongbe DO, Adeagbo AO. Cigarette Smoking Practices and Its Determinants Among University Students in Southwest, Nigeria. *Journal of Asian Scientific Research.* 2012;2(2):62-69.

18. Dania MG, Ozoh OB, Bandele EO. Smoking habits, awareness of risks, and attitude towards tobacco control policies among medical students in Lagos, Nigeria. *Annals of African medicine.* 2015;14(1):1-7.

19. Desalu O, Olokoba A, Danburam A, Salawu F, Issa B. Epidemiology of tobacco smoking among adults population in north-east Nigeria. *The internet journal of epidemiology.* 2008;6(1).

20. Desalu OO, Salami AK, Fawibe AE. Prevalence of cough among adults in an urban community in Nigeria. *West African journal of medicine.* 2011;30(5):337-341.

21. Ebirim CIC, Amadi AN, Abanobi OC, Iloh GUP. The prevalence of cigarette smoking and knowledge of its health implications among adolescents in Owerri, South-Eastern Nigeria. *Health.* 2014;6(12):1532-1538.

22. Ekanem US, Opara DC, Akwaowo CD. High blood pressure in a semi-urban community in south-south Nigeria: a community-based study. *African health sciences.* 2013;13(1):56-61.

23. Emerole CO, Aguwa EN, Onwasigwe CN, Nwakoby BA. Cardiac risk indices of staff of Federal University Of Technology Owerri, Imo State, Nigeria. *Tanzania health research bulletin.* 2007;9(2):132-135.

24. Fatoye FO, Morakinyo O. Substance use amongst secondary school students in rural and urban communities in south western Nigeria. *East African medical journal.* 2002;79(6):299-305.

25. Fawibe A, Shittu A. Prevalence and characteristics of cigarette smokers among undergraduates of the University of Ilorin, Nigeria. *Nigerian journal of clinical practice.* 2011;14(2):201-205.

26. Hussain NA, Akande TM, Adebayo O. Prevalence of cigarette smoking and the knowledge of its health implications among Nigerian soldiers. *East African journal of public health.* 2009;6(2):168-170.

27. Ibekwe R. Modifiable Risk factors of Hypertension and Socio-demographic Profile in Oghara, Delta State; Prevalence and Correlates. *Annals of medical and health sciences research.* 2015;5(1):71-77.

28. Makanjuola AB, Daramola TO, Obembe AO. Psychoactive substance use among medical students in a Nigerian university. *World psychiatry : official journal of the World Psychiatric Association (WPA).* 2007;6(2):112-114.

29. Morakinyo J, Odejide AO. A community based study of patterns of psychoactive substance use among street children in a local government area of Nigeria. *Drug and alcohol dependence.* 2003;71(2):109-116.

30. Obot IS. The use of tobacco products among Nigerian adults: a general population survey. *Drug and alcohol dependence.* 1990;26(2):203-208.

31. Odey FA, Okokon IB, Ogbeche JO, Jombo G, Ekanem E. Prevalence of cigarette smoking among adolescents in Calabar city, south-eastern Nigeria. *Journal of Medicine and Medical Sciences.* 2012;3(4):237-242.

32. Odeyemi KA, Osibogun A, Akinsete AO, Sadiq L. The Prevalence and Predictors of Cigarette Smoking among Secondary School Students in Nigeria. *The Nigerian postgraduate medical journal.* 2009;16(1):40-45.

33. Odugbemi TO, Onajole AT, Osibogun AO. Prevalence of cardiovascular risk factors amongst traders in an urban market in Lagos, Nigeria. *The Nigerian postgraduate medical journal.* 2012;19(1):1-6.

34. Lawoyin TO, Asuzu MC, Kaufman J, et al. Prevalence of cardiovascular risk factors in an African, urban inner city community. *West African journal of medicine.* 2002;21(3):208-211.

35. Ige OK, Owoaje ET, Adebiyi OA. Non communicable disease and risky behaviour in an urban university community Nigeria. *African health sciences.* 2013;13(1):62-67.

36. Ugwuja E, Ogbonna N, Nwibo A, Onimawo I. Overweight and Obesity, Lipid Profile and Atherogenic Indices among Civil Servants in Abakaliki, South Eastern Nigeria. *Annals of medical and health sciences research.* 2013;3(1):13-18.

37. Odukoya OO, Odeyemi KA, Oyeyemi AS, Upadhyay RP. Determinants of smoking initiation and susceptibility to future smoking among school-going adolescents in Lagos State, Nigeria. *Asian Pacific journal of cancer prevention : APJCP.* 2013;14(3):1747-1753.

38. Okagua J, Opara P, Alex-Hart BA. Prevalence and determinants of cigarette smoking among adolescents in secondary schools in Port Harcourt, Southern Nigeria. *International journal of adolescent medicine and health.* 2016;28(1):19-24.

39. Oladapo OO, Salako L, Sodiq O, Shoyinka K, Adedapo K, Falase AO. A prevalence of cardiometabolic risk factors among a rural Yoruba south-western Nigerian population: a population-based survey. *Cardiovascular journal of Africa.* 2010;21(1):26-31.

40. Onofa L. *Prevalence and pattern of drug abuse among students of three tertiary institutions in Abeokuta*, A Dissertation submitted to the West African College of Physicians, Faculty of Psychiatry; 2006.

41. Onyeonoro UU, Chukwuonye II, Madukwe OO, Ukegbu AU, Akhimien MO, Ogah OS. Awareness and perception of harmful effects of smoking in Abia State, Nigeria. *Nigerian Journal of Cardiology.* 2015;12(1):27.

42. Oshodi OY, Aina OF, Onajole AT. Substance use among secondary school students in an urban setting in Nigeria: prevalence and associated factors. *African journal of psychiatry.* 2010;13(1):52-57.

43. Owonaro P, Eniojukan J. Cigarette Smoking Practices, Perceptions and Awareness of Government Policies among Pharmacy Students in Niger Delta University in South-South Nigeria. *UK Journal of Pharmaceutical and Biosciences.* 2015;3(5):20-29.

44. Owonaro P, Eniojukan J. The Prevalence and Contextual Correlates of Smoking in Opokuma Clan of Bayelsa State, Nigeria. *International Journal of Advances in Pharmacy, Biology and Chemistry.* 2015;4(3):656-667.

45. Ozoh OB, Dania MG, Irusen EM. The Prevalence of Self-Reported Smoking and Validation with Urinary Cotinine Among Commercial Drivers in Major Parks in Lagos, Nigeria. *Journal of public health in Africa.* 2014;5(1):316.

46. Ozoh OB, Akanbi MO, Amadi CE, Vollmer W, Bruce N. The prevalence of and factors associated with tobacco smoking behavior among long-distance drivers in Lagos, Nigeria. *African health sciences.* 2017;17(3):886-895.

47. Raji M, Abubakar I, Oche M, Kaoje A. Prevalence and determinants of cigarette smoking among in school adolescents in Sokoto metropolis, Northwest Nigeria. *International Journal of Tropical Medicine.* 2013;8(3):81-86.

48. Raji M, Usman A, Umar M, Oladigbolu R, Kaoje A. Cigarette Smoking among Out-of-School Adolescents in Sokoto Metropolis, North-West Nigeria. *Health Science Journal.* 2017;11(3).

49. Salawu F, Danburam A, Isa B, Agbo J. Cigarette smoking habits among adolescents in northeast Nigeria. *Internet J Epidemiol.* 2010;8(1):1.

50. Shehu AU, Idris SH. Marijuana smoking among secondary school students in Zaria, Nigeria: factors responsible and effects on academic performance. *Annals of African medicine.* 2008;7(4):175-179.

51. Uwakwe R, Gureje O. Sociodemographic correlates of continuing tobacco use - a descriptive report from the Nigerian Survey of Mental Health and Wellbeing. *Acta psychiatrica Scandinavica.* 2016;133(6):506-513.

52. Yisa IO, Lawoyin TO, Fatiregun AA, Emelumadu OF. Pattern of substance use among senior students of command secondary schools in Ibadan, Nigeria. *Nigerian journal of medicine : journal of the National Association of Resident Doctors of Nigeria.* 2009;18(3):286-290.

53. Abasiubong F, Atting I, Bassey E, Ekott J. A comparative study of use of psychoactive substances amongst secondary school students in two local Government Areas of Akwa Ibom State, Nigeria. *Nigerian journal of clinical practice.* 2008;11(1):45-51.

54. Gureje O, Degenhardt L, Olley B, et al. A descriptive epidemiology of substance use and substance use disorders in Nigeria during the early 21st century. *Drug and alcohol dependence.* 2007;91(1):1-9.

55. Lasebikan VO, Ola BA. Prevalence and Correlates of Alcohol Use among a Sample of Nigerian Semirural Community Dwellers in Nigeria. 2016;2016:2831594.

56. Odenigbo CU, Oguejiofor OC, Odenigbo UM, Ibeh CC, Ajaero CN, Odike MA. Prevalence of dyslipidaemia in apparently healthy professionals in Asaba, South South Nigeria. *Nigerian journal of clinical practice.* 2008;11(4):330-335.

57. Forrest KY, Bunker CH, Kriska AM, Ukoli FA, Huston SL, Markovic N. Physical activity and cardiovascular risk factors in a developing population. *Medicine and science in sports and exercise.* 2001;33(9):1598-1604.

58. Oguoma VM, Nwose EU, Skinner TC, Digban KA, Onyia IC, Richards RS. Prevalence of cardiovascular disease risk factors among a Nigerian adult population: relationship with income level and accessibility to CVD risks screening. *BMC public health.* 2015;15:397.

59. Ezejimofor MC, Uthman OA, Maduka O, et al. The Burden of Hypertension in an Oil- and Gas-Polluted Environment: A Comparative Cross-Sectional Study. *American journal of hypertension.* 2016;29(8):925-933.

60. Ezekwesili CN, Ononamadu CJ, Onyeukwu OF, Mefoh NC. Epidemiological survey of hypertension in Anambra state, Nigeria. *Nigerian journal of clinical practice.* 2016;19(5):659-667.

61. Ogah OS, Madukwe OO, Chukwuonye, II, et al. Prevalence and determinants of hypertension in Abia State Nigeria: results from the Abia State Non-Communicable Diseases and Cardiovascular Risk Factors Survey. *Ethnicity & disease.* 2013;23(2):161-167.

62. Abdur-Rahman LO, Baba S, Bamigbola KT, et al. Outcome of management of complicated extragonadal teratoma in a resource poor setting. *African journal of paediatric surgery : AJPS.* 2013;10(4):323-326.

63. Ugwuja E, Ezenkwa U, Nwibo A, Ogbanshi M, Idoko O, Nnabu R. Prevalence and determinants of hypertension in an agrarian rural community in southeast Nigeria. *Annals of medical and health sciences research.* 2015;5(1):45-49.

64. Wahab KW, Okokhere PO, Ugheoke AJ, Oziegbe O, Asalu AF, Salami TA. Awareness of warning signs among suburban Nigerians at high risk for stroke is poor: a cross-sectional study. *BMC neurology.* 2008;8:18.
